# Site-specific ubiquitination of MLKL targets it to endosomes and targets *Listeria* and *Yersinia* to the lysosomes

**DOI:** 10.1038/s41418-021-00924-7

**Published:** 2022-01-09

**Authors:** Seongmin Yoon, Konstantin Bogdanov, David Wallach

**Affiliations:** Department of Biomolecular Sciences, The Weizmann Institute of Science, 7610001 Rehovot, Israel

**Keywords:** Mechanism of action, Signal transduction

## Abstract

Phosphorylation of the pseudokinase mixed lineage kinase domain-like protein (MLKL) by the protein kinase RIPK3 targets MLKL to the cell membrane, where it triggers necroptotic cell death. We report that conjugation of K63-linked polyubiquitin chains to distinct lysine residues in the N-terminal HeLo domain of phosphorylated MLKL (facilitated by the ubiquitin ligase ITCH that binds MLKL via a WW domain) targets MLKL instead to endosomes. This results in the release of phosphorylated MLKL within extracellular vesicles. It also prompts enhanced endosomal trafficking of intracellular bacteria such as *Listeria monocytogenes* and *Yersinia enterocolitica* to the lysosomes, resulting in decreased bacterial yield. Thus, MLKL can be directed by specific covalent modifications to differing subcellular sites, whence it signals either for cell death or for non-deadly defense mechanisms.

## Introduction

Exploration of the mechanisms by which cytokines of the TNF family induce cell death has led to the identification of a form of programmed cell death called “necroptosis” in which the protein mixed lineage kinase domain-like molecule (MLKL), upon its phosphorylation by the protein kinase RIPK3, triggers rupture of the cellular membrane. This pathway was later shown also to be activated by a number of other inducers, including other cytokines, as well as by various pathogen components. Differing inducing agents activate RIPK3 via the functioning of different upstream molecules (the protein kinase RIPK1, the adapter protein TRIF or the nucleic acid sensor DAI/ZBP1), all of which associate with RIPK3 via a common motif that they share with it, called the “RHIM domain” [1–7].

MLKL itself is a pseudokinase with an evolutionarily conserved, N-terminal four α-helical bundle domain called the “HeLo domain,” which mediates cell death. RIPK3 activates MLKL by phosphorylating it in the region homologous to the kinase-activating loop. The exact mechanism of death mediation by MLKL is still unclear, other than that it is initiated by a conformational change in MLKL that results in its oligomerization and also in the exposure of clusters of lipid-binding epitopes within its HeLo domain. The ensuing death appears to be inflicted by the binding of the exposed residues to certain lipids in the cell membrane. Association of some of these residues with phosphatidylinositol-phosphate phospholipids was suggested to prompt a further conformational change in MLKL, endowing it with the ability to rupture the cell membrane [8–10]. Detailed mutational and structural analyses of the HeLo domains of human and mouse MLKL revealed evolutionary divergence, reflected in the involvement of different epitopes in these two species in death mediation, at differing locations in their HeLo domains and with differing abilities to bind lipids. However, studies of both species have so far yielded no clear notion of the way by which association of these epitopes with the cellular membrane dictates its rupture [reviewed in [9]. Also see 10.1101/2021.05.03.442385].

Both the activation of MLKL and the exertion of its deadly function are subject to regulation by a variety of different mechanisms. Those most thoroughly studied are mechanisms that modulate the activation of MLKL by receptors of the TNF family, where three signaling proteins that these receptors activate—the ubiquitin ligases cIAP1 and cIAP2 and the protease caspase-8—act to withhold induction of necroptotic death via MLKL and instead to initiate other functional changes—gene activation, or induction of apoptotic death. Besides the modulation of MLKL activation as a result of modifications of RIPK1 by the cIAPs or caspase-8, we know of various other induced modifications of the proteins that act upstream of MLKL, as well as of MLKL itself, which modulate the functions of each of these proteins, and are dictated by quite a large number of different proteins that bind to them [11].

Recent studies have revealed that not only the effectiveness of MLKL activation but also the nature of the function that it serves is subject to modulation. It was found, for example, that MLKL mediates activation of the NLRP3 inflammasome with the resulting generation of inflammatory cytokines and their release by the cell, and that this occurs in certain cells at a time that death mediation by MLKL has been arrested [12, 13]. MLKL was also found to promote regeneration of axons by facilitating the breakdown of their myelin sheaths [14], and to enhance the expression of endothelial cell adhesion proteins by stabilizing their mRNAs [15]. Proteins containing HeLo domains in plant cells and in yeasts were also found both to mediate cell death and to initiate various non-deadly functional changes in cells [8, 16–19].

Alongside the emerging awareness of the heterogeneity of MLKL functions, there is growing knowledge of heterogeneous subcellular sites of action of this protein. Most of the MLKL in cells appears to occur prior to stimulation in the cytoplasm, from where part of it translocates, upon activation, to the cell membrane. However, MLKL has also been discerned in the nucleus [20, 21], where it serves some non-deadly functions [15] and from where it can also translocate to the cell membrane and mediate the induction of necroptotic cell death there [20–22].

MLKL also associates with endosomal membranes, from where it is released from the cell within extracellular vesicles (EVs) [23, 24]. Besides mediating MLKL release from the cell, the association of MLKL with endosomes facilitates endosomal trafficking. Up to now, the physiological function served by this enhancement, and the signals dictating such association of MLKL with the endosomal membranes, were unknown [23]. We now show that the endosomal association of MLKL is dictated by the linkage of polyubiquitin chains to distinct lysine residues in its HeLo domain. We also show that the enhancement of endosomal trafficking by polyubiquitin-conjugated MLKL molecules contributes to the defense against pathogenic bacteria by facilitating their destruction in the lysosomes.

## Results

### Activation of MLKL triggers its K63-linked ubiquitination at specific lysine residues

**Fig. 1:**
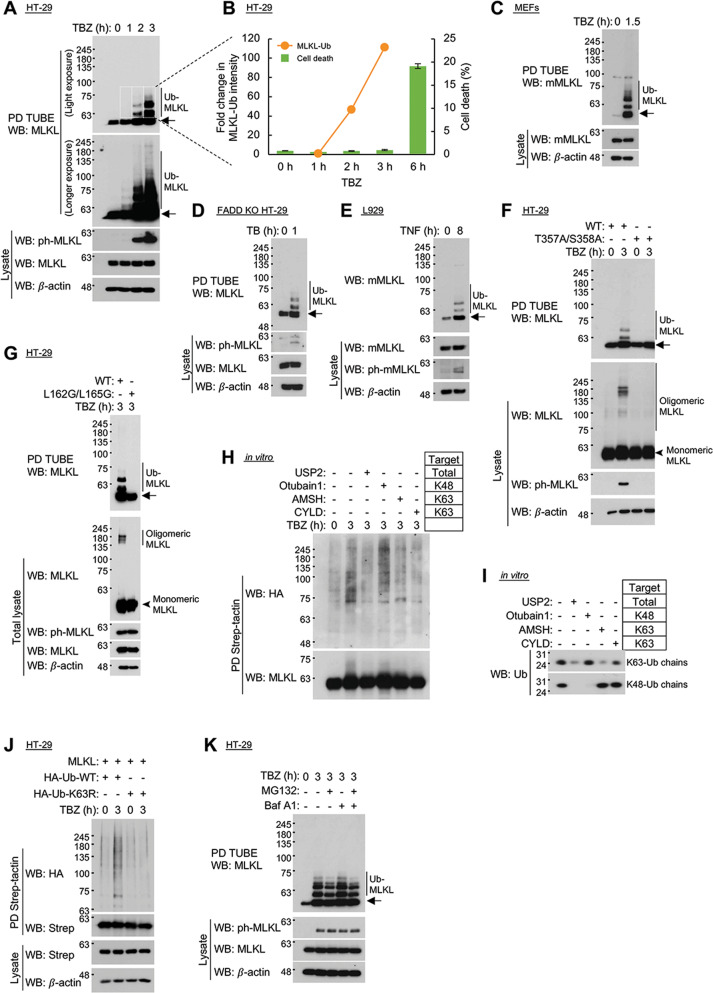
Activation of MLKL triggers its K63-linked ubiquitination. **A**, **B** Time-dependent MLKL ubiquitination and phosphorylation in human HT-29 cells in response to treatment with TBZ (TNF, 1000 U/ml, the bivalent IAP antagonist BV6, 1 μM, and the caspase inhibitor z-VAD-fmk, 20 μM. These are the concentrations at which these three reagents were applied in all other experiments). **A** Western blot analysis (WB) of 15 μg of the proteins of the total cellular lysates (bottom) and of the proteins pulled down (PD) from these amounts of lysates using TUBE1-agarose (top). Unless otherwise specified, arrows pointing to western blots that were probed with anti-MLKL antibodies indicate non-ubiquitinated MLKL. **B** Comparison of the extents of ubiquitination and of cell death at different time points. Cell death was assessed twice in duplicates (*n* = 4), 6with practically identical results. **C**–**E** Assessments of MLKL ubiquitination and phosphorylation: in **C** MEFs in response to TBZ; in **D** HT-29 cells in which FADD was knocked out by CRISPR/Cas9 in response to treatment with just TB, i.e., TNF (1000 U/ml) and Bv6 (1 μM); and in **E** L929 cells in response to TNF alone (1000 U/ml in this and in all other experiments). **F**, **G** MLKL ubiquitination in response to TBZ depends on its phosphorylation and oligomerization. The extents of MLKL ubiquitination (top), aggregation (middle) and phosphorylation (bottom) were determined in MLKL-knockout (KO) HT-29 cells constitutively expressing wild-type MLKL and **F** a non-phosphorylatable MLKL mutant (T357A/S358A), or **G** the coiled-coil domain mutant (L162G/L165G). **H** Comparison of the in vitro effects of the indicated deubiquitinases on HA-tagged polyubiquitin chains that were conjugated to MLKL in HT-29 cells by their treatment for 3 h with TBZ. **I** Effects of these deubiquitinases on synthetic K63-linked and K48-linked ubiquitin chains. **J** Conjugation of wild-type and K63R-mutated HA-tagged ubiquitin to MLKL in HT-29 cells. **K** Accumulation of ubiquitinated MLKL in response to TBZ is not enhanced by treatment for 3 h with MG132 (10 μM) or bafilomycin A1 (Baf A1, 0.1 μM).

Besides phosphorylating MLKL, treatment of cells by agents that activate the protein kinase RIPK3 triggers a time-dependent MLKL ubiquitination [25]. We found that such ubiquitination could be induced in human epithelial HT-29 cells by combined treatment with the cytokine TNF, the inhibitor of apoptosis proteins (IAP) antagonist BV6, and the caspase inhibitor z-VAD-fmk (TBZ). The ubiquitination correlated to the induction of MLKL phosphorylation and could be blocked by the RIPK3 inhibitor GSK-872. Kinetic analysis revealed that a high extent of ubiquitination was reached in the HT-29 cells several hours before any sign of cell death could be observed (Fig. 1A, B and Supplementary Fig. 2). Such ubiquitination could also be induced by TBZ in mouse embryonic fibroblasts (MEFs; Fig. 1C). In HT-29 cells in which caspase activation by TNF was compromised by knockout (KO) of the adapter protein FADD, ubiquitination could be induced by treatment with just TNF and BV6 (Fig. 1D), whereas in cells of the L929 line that are derived from mouse fibroblasts, MLKL ubiquitination, similarly to its phosphorylation and necroptotic death [26], could be induced by treatment with TNF alone (Fig. 1E).

While assessing the impact of various mutations in human MLKL on its ubiquitination, we found that the ubiquitination was prevented by a mutation that ablates the phosphorylation of MLKL (T357A/S358A [4], Fig. 1F), while not affecting its recruitment to RIPK3 (Supplementary Fig. 3). It was also prevented by a mutation that affects neither the recruitment of MLKL to RIPK3 nor the phosphorylation of MLKL, but compromises its oligomerization (L162G/L165G [8], Fig. 1G and Supplementary Fig. 3). These findings suggested that, like the necroptotic effect of MLKL, its ubiquitination depends on its induced oligomerization.

The MLKL-conjugated ubiquitin chains could be degraded by the K63-specific deubiquitinases CYLD and AMSH, as well as by the broad-specificity deubiquitinase USP2, but not by the K48-specific deubiquitinase Otubain1 (Fig. 1H, I). Comparison between the effectiveness of incorporation of the wild-type (WT) and incorporation of the K63R mutant ubiquitin to the MLKL-conjugated ubiquitin chains suggested that the ubiquitin moieties in these chains are indeed interlinked via their K63 residues (Fig. 1J).

While K48-linked ubiquitination of proteins serves mostly to dictate their proteolytic degradation in the proteasome, K63-linked ubiquitination often serves other roles [27]. Consistently, we found that the application of a proteasomal inhibitor to TBZ-treated cells did not result in increased amounts of ubiquitinated MLKL. Nor was such an increase observed in cells treated with Bafilomycin A1, which arrests lysosomal degradation (Fig. 1K).

**Fig. 2:**
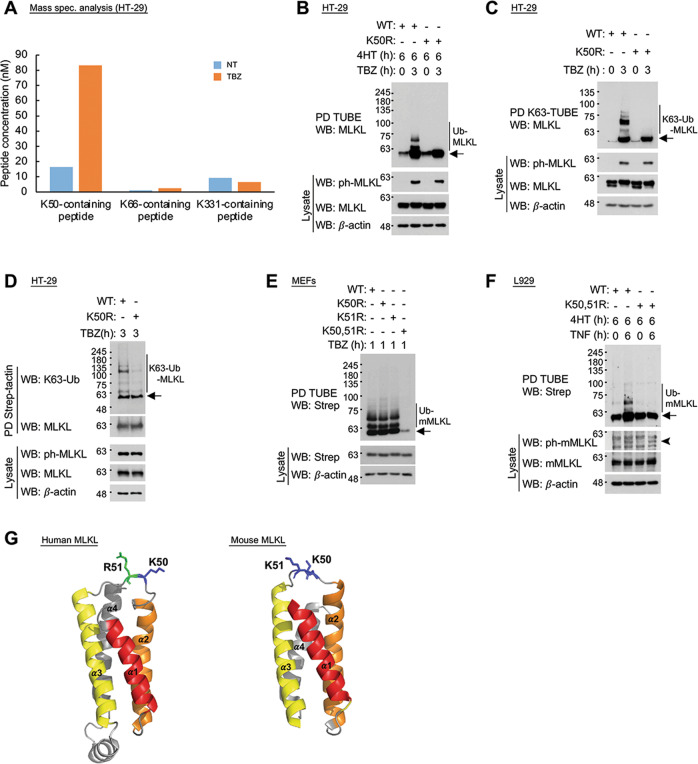
MLKL is ubiquitinated at specific lysine residues. **A** Results of quantitative mass spectroscopic analysis of MLKL ubiquitination in HT-29 cells and of its increase by treatment for 3 h with TBZ. The K50-containing peptide is VLGLIKPLEMLQDQGKR, the K66-containing peptide is FKAALEEANGEIEK and the K331-containing peptide is LHHSEAPELHGKIR. Both in untreated and in TBZ-treated cells, the amounts of K50-containing peptide in which the methionine residue was oxidized were less than 1% of the total amounts of this peptide. **B** Assessment of MLKL ubiquitination in TBZ-treated HT-29 cells in which endogenous MLKL had been knocked out, and either the wild-type or the K50R-mutated human MLKL was inducibly re-expressed under control of the GEV16/pF5x UAS system. **C** TBZ-treated MLKL KO HT-29 cells that inducibly express wild-type MLKL, but not cells expressing the K50R MLKL mutant, contain ubiquitinated MLKL molecules that bind K63-TUBE (a reagent that specifically recognizes K63-linked polyubiquitin chains). **D** K63-linked polyubiquitination of wild-type MLKL, but not of its K50R mutant, can be discerned by an antibody that specifically recognizes the K63 link. Western blot analysis was done using MLKL preparations that were affinity-purified from lysates of TBZ-treated HT-29 cells, as described in Materials and methods. **E** Assessment of MLKL ubiquitination in TBZ-treated MLKL KO MEFs expressing the indicated mutants of mouse MLKL as in **B**. **F** Assessment of MLKL ubiquitination in TNF-treated MLKL KO L929 cells expressing either wild-type or K50,51R-mutated mouse MLKL as in **B**. The arrowhead points to phosphorylated mouse MLKL. **G** Site of MLKL ubiquitination within the HeLo domain structure. Shown are ribbon diagrams of the HeLo domains in human MLKL (amino acids 4–120) and mouse MLKL (amino acids 2−115), drawn using PyMOL software based on the solution structure of the human MLKL N-terminal region, as determined using nuclear magnetic resonance [65] (PDB 2MSV), and the crystal structure of full-length mouse MLKL, as determined by X-ray diffraction [66] (PDB 4BTF). The helices forming the four-helix bundle are denoted and colored, as are the lysine residues that we found to be ubiquitinated (blue) and arginine 51 in human MLKL (green).

In mass spectroscopy analysis of MLKL that was affinity-purified from HT-29 cells that were treated for 3 h with TBZ, we discerned, in two independent experiments, ubiquitination of K50, K66, and K331 in MLKL (Supplementary Fig. 4). Subsequent quantitative analysis revealed that the ubiquitination was highest in K50, and that upon TBZ treatment it increased dramatically. In contrast, the limited ubiquitination of K331 decreased upon TBZ treatment. The extent of ubiquitination of K66 was very low compared to that of either K50 or K331 (Fig. 2A).

To further verify that K50 is a major site of ubiquitination in MLKL, we knocked out MLKL in HT-29 cells by CRISPR/Cas9 gene editing, then inducibly expressed in them either WT MLKL or an MLKL mutant in which lysine 50 was replaced with arginine (K50R), and then assessed the ubiquitination of both proteins upon TBZ treatment. As shown in Fig. 2B, the K50R mutation greatly reduced the ubiquitination of MLKL. The polyubiquitin chains linked to K50 in MLKL could be shown, both by application of agarose K63-TUBE or of an antibody specific to the K63 link between ubiquitin chains, to be K63-conjugated (Fig. 2C, D).

The site in mouse MLKL that is homologous to the site of ubiquitination in human MLKL possesses two adjacent lysine residues, at positions 50 and 51. Mutation of the lysine residue at position 50 in MEFs did not affect the extent of its TBZ-induced ubiquitination, nor was the ubiquitination affected by mutation of the neighboring lysine residue at position 51. However, both in MEFs and in the mouse L929 cells, ubiquitination was dramatically reduced when lysines 50 and 51 were each replaced by arginine (Fig. 2E, F).

According to a recently published study, mouse MLKL is also ubiquitinated at lysine 219, and it was suggested that this ubiquitination, as well as ubiquitination of the corresponding lysine residue in human MLKL (at position 230), serve to facilitate death induction [28]. However, in our repeated mass spectroscopy analysis we could not discern ubiquitination of lysine 230 in HT-29 cells that had been treated for 3 h with TBZ. Moreover, the replacement of lysine 230 with arginine slightly enhanced death induction in these cells (Supplementary Fig. 6A). The decrease in the effectiveness of death induction that was previously reported to occur when replacing this residue with methionine [29] is thus not due to the arrest of ubiquitination, but rather reflects an impact of an altered local charge in the MLKL molecule on its function.

On assessing the effects of replacing lysine 50 in human MLKL and lysines 50 and 51 in mouse MLKL on cell death induction, we found that both resulted in some enhancement of the cytotoxic activity of MLKL, and that its extent varied from one cell type to another (Supplementary Fig. 6B, C). Such increase was observed when this necroptotic death was induced not only by TNF but also by stimulation of TLR3, which employs TRIF rather than RIPK1 to activate the necroptotic pathway [30], and in that case too, mutation of lysines 50 and 51 compromised the induced ubiquitination of MLKL (Supplementary Fig. 7).

As shown in Fig. 2G, both in human and in mouse MLKL molecules, these lysine residues occur in the loop connecting helices α2 and α3 in the HeLo domain. Prior studies had suggested that in the case of human MLKL, this region contributes to the association of the molecule with phosphatidylinositol-phosphate phospholipids and may thus affect its activation [31, 32]. In mouse MLKL, however, this region does not seem to serve such a role and is quite remote from the residues shown to be crucially involved in the mediation of necroptosis ([33] and 10.1101/2021.05.03.442385). It, therefore, seemed to us plausible that the observed modulation of the effectiveness of necroptosis by MLKL ubiquitination reflects a functional role(s) of such ubiquitination that is different from its direct involvement in the necroptotic effect.

### Ubiquitination of MLKL prompts its association with endosomal membranes and its exclusion from cells within extracellular vesicles

**Fig. 3:**
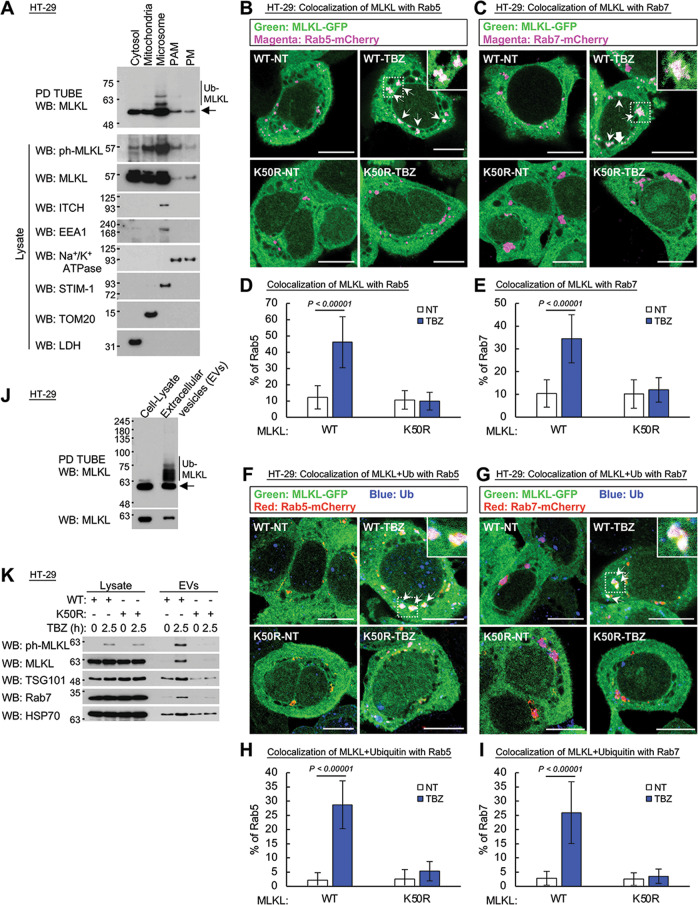
Ubiquitinated MLKL associates with endosomal membranes and is excluded from the cell within extracellular vesicles. **A** Assessment of the occurrence of ubiquitinated and phosphorylated MLKL, induced by treatment for 3 h with TBZ, in the indicated subcellular fractions of HT-29 cells. Samples of 15 μg per well of the proteins in the lysates of each fraction were subjected to western blot analysis, and samples of 170 μg per well of the proteins were assessed for MLKL ubiquitination. **B**–**I** Microscopic analysis of the effect of the K50R mutation in MLKL on the association of MLKL (**B**−**E**) and of both MLKL and ubiquitin (**F**−**I**) with the early and late endosomes (visualized by constitutive expression of Rab5 or Rab7 fused to mCherry) in MLKL KO HT-29 cells inducibly expressing MLKL or its K50R mutant fused to GFP, observed after treatment for 2.5 h with TBZ. Ubiquitin was detected by immunostaining with its specific antibody. **B**, **C**, **F**, **G** Examples of the fluorescence images. **D**, **E**, **H**, **I** Quantification of the data (~110 cells). Arrows point to MLKL-GFP + Rab5 or Rab7 fused to mCherry in **B** and **C**, and to MLKL-GFP + Rab5 or Rab7 fused to mCherry + ubiquitin in **F** and **G**. Scale bar, 10 μm. **J** Comparison of the amounts of MLKL and of ubiquitinated MLKL in 60 μg of proteins of the total cellular lysate and of EVs released from HT-29 cells during treatment for 3 h with TBZ. **K** Effect of the K50R mutation in MLKL on MLKL release in EVs from HT-29 cells treated for 2.5 h with TBZ. Samples of 15 μg of cellular lysates and of EVs derived from 4 × 10^7^ cells were applied to each lane.

In comparing the amounts of ubiquitinated MLKL in various fractions of TBZ-treated HT-29 cells, we found it to be enriched in the microsomal membranes and very low in the cytosol (Fig. 3A). Consistently, some of the MLKL molecules in HT-29 cells were found to associate with isolated endosomal membranes and to co-localize with Rab5 and Rab7, markers of early and late endosomes, respectively. This association underwent a dramatic increase following TBZ treatment (Fig. 3B−E and Supplementary Fig. 8).

Staining with anti-ubiquitin antibodies revealed co-association of ubiquitin and MLKL with the endosomes (which was similarly increased in response to TBZ treatment (Fig. 3F−I)), but not with the endoplasmic reticulum or the mitochondria (Supplementary Fig. 9 and data not shown). The association of ubiquitin with MLKL in the endosomes was confirmed by bimolecular fluorescence complementation (BiFC) analysis (Supplementary Fig. 10). Strikingly, HT-29 cells in which lysine 50 in MLKL was mutationally replaced with arginine (K50R) did not display these associations with the endosomal membranes (Fig. 3B−I and Supplementary Fig. 8).

We previously reported that the binding of MLKL to endosomal membranes eventually results in the exclusion of these MLKL molecules from the cells within EVs [23]. As shown in Fig. 3J, MLKL molecules found in the EVs are highly ubiquitinated, and to an extent much greater than those found intracellularly. The arrest of the ubiquitination of MLKL by the K50R mutation practically abolished its exclusion within EVs (Fig. 3K). These findings suggested that both the association of MLKL with the endosomes and its eventual exclusion within EVs are dictated by its ubiquitination.

### MLKL ubiquitination is mediated by ITCH

**Fig. 4:**
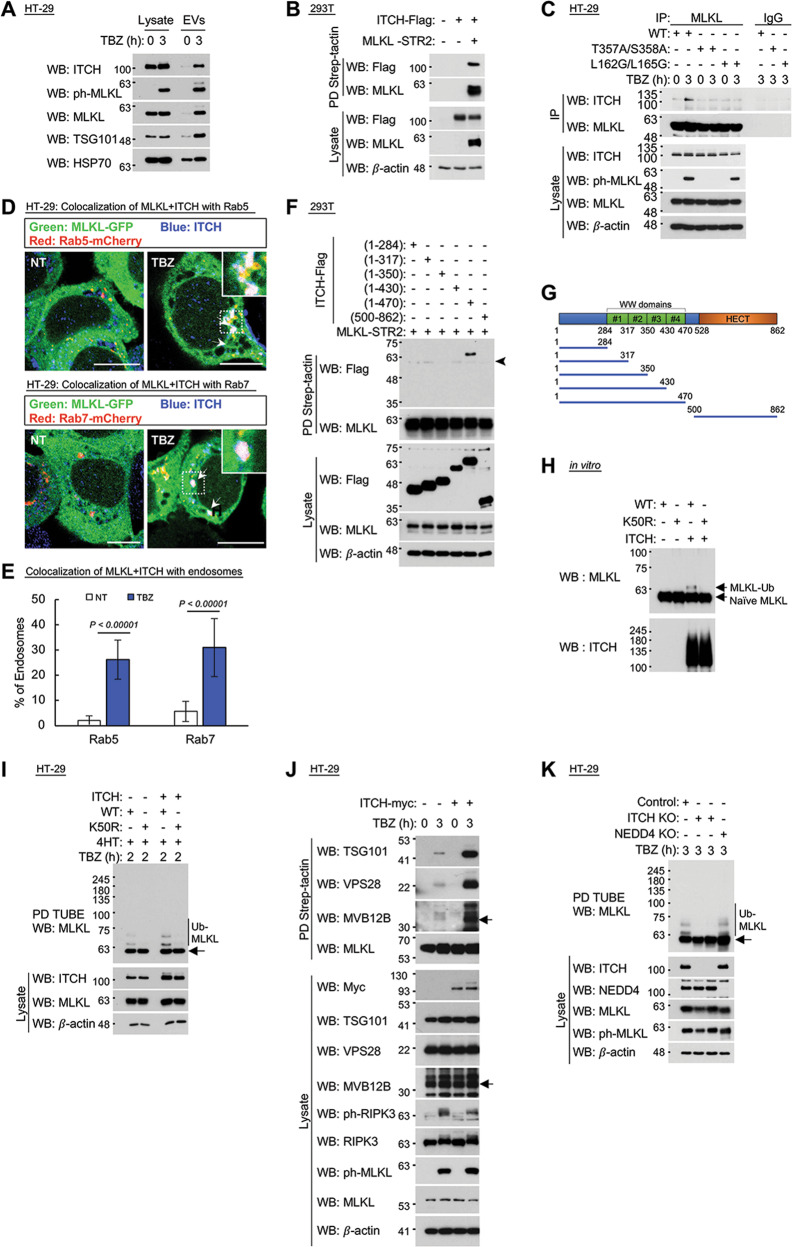
MLKL ubiquitination is mediated by ITCH. **A** Comparison of the amounts of ITCH in 15 μg of proteins of the total cellular lysate and of EVs released from HT-29 cells during treatment for 3 h with TBZ. **B** ITCH binds to MLKL in HEK293T cells transiently overexpressing the two proteins. Samples were subjected to western blot analysis following precipitation of MLKL via a fused Strep-tag. **C** In response to TBZ, ITCH binds inducibly to MLKL in MLKL knocked-down HT-29 cells constitutively expressing wild-type MLKL, but not to its T357A/S358A or L162G/L165G mutants. MLKL (wild-type and mutants) were precipitated using an anti-MLKL antibody (ab184718 from Abcam). **D**, **E** Immunocytochemical evidence for the colocalization of ITCH and activated MLKL in association with endosomes. **D** Immunofluorescence images. Arrows point to MLKL-GFP + Rab5 or 7 fused to mCherry + ITCH. ITCH was detected by immunostaining with its specific antibody. Scale bar, 10 μm. **E** Quantification of the data (75 cells). **F**, **G** Deletion analysis for identification of the region in the ITCH molecule to which MLKL binds. **F** Western blot analysis of the binding of various ITCH deletion mutants to MLKL fused C-terminally to the double Strep-tag upon their transient expression in HEK293T cells. MLKL was eluted by biotin after its precipitation by Strep-Tactin beads. The arrowhead points to non-specific bands. **G** Diagram showing the deletion mutants used and the location of WW motifs in ITCH. **H** Recombinant ITCH catalyzes the in vitro ubiquitination of MLKL isolated from HT-29 cells, but not of its K50R mutant. **I** Enhancement of TBZ-induced ubiquitination of MLKL, but not of its K50R mutant, when inducibly expressed in MLKL KO HT-29 cells, by inducibly expressed ITCH. **J** Enhancement of the association of MLKL (expressed inducibly in MLKL KO HT-29 cells) with ESCRT proteins, in response to TBZ treatment, by transient expression of ITCH. (Arrows indicate MVB12B.) **K** TBZ-induced MLKL ubiquitination in HT-29 cells is compromised by CRISPR/Cas9-facilitated KO of ITCH, but not by KO of NEDD4. Two distinct clones of HT-29 ITCH KO were analyzed, with identical results.

In seeking a clue to the mechanism by which MLKL is ubiquitinated, we re-examined the list of proteins previously found on our mass spectroscopic analysis to associate with MLKL within the EVs released from TBZ-treated HT-29 cells [23]. We noticed that the list included the ubiquitin ligase ITCH [34, 35], and western blot analysis confirmed that TBZ treatment of HT-29 cells indeed induces the release of ITCH within EVs, alongside MLKL (Fig. 4A).

Further evidence for a direct association of the two proteins came from the finding that when ITCH and MLKL were overexpressed in HEK293T cells, they effectively bound to each other (Fig. 4B). At their endogenous cellular levels, the two proteins did not associate in non-stimulated cells. However, they bound together effectively following the triggering of MLKL phosphorylation by TBZ. Such association was also observed in cells expressing the K50R MLKL mutant, but not in cells expressing MLKL mutants that had failed to be phosphorylated (T357A/S358A) or to oligomerize following phosphorylation (L162G/L165G) (Fig. 4C and Supplementary Fig. 12).

Immunostaining of HT-29 cells showed that following TBZ treatment, MLKL colocalizes with ITCH in both the early and the late endosomes (Fig. 4D, E). In deletion analysis, we found that MLKL binds ITCH via its pseudokinase motif (Supplementary Fig. 13) and that, characteristically of HECT-type E3 ligases, ITCH binds MLKL via a WW motif (Fig. 4F, G).

In in vitro tests, we found that recombinant ITCH can bind both to human and to mouse recombinant MLKL and ubiquitinates them (Supplementary Fig. 14), and that although ITCH catalyzes the ubiquitination of WT MLKL, it does not ubiquitinate the MLKL’s K50R mutant (Fig. 4H). Consistently, overexpression of ITCH in TBZ-treated HT-29 cells enhanced the ubiquitination of MLKL, but not of K50R MLKL (Fig. 4I). Overexpression of ITCH also dramatically enhanced the association of MLKL with ESCRT proteins (Fig. 4J), which apparently dictate the association of MLKL with the endosomes and its eventual release from the cells in EVs [23].

Conversely, the KO of ITCH in HT-29 cells by CRISPR/Cas9 gene editing compromised this ubiquitination. KO of NEDD4, another HECT-type E3 ligase that also occurs in the HT-29 cells, had no effect on MLKL ubiquitination (Fig. 4K).

Taken together, these findings suggested that ITCH is the E3 ligase that mediates ubiquitination of the HeLo domain in MLKL and thus dictates the association of MLKL with endosomes. Consistently with the observed increase in necroptotic activity of the non-ubiquitinable K50R mutant (Supplementary Fig. 6B, C), KO of ITCH—the enzyme mediating this ubiquitination—was found to somewhat augment the induction of necroptotic death (Supplementary Fig. 15).

### MLKL ubiquitination facilitates the destruction of intracellular *Listeria monocytogenes* in a manner distinct from the reported direct cytotoxic effect of MLKL on these bacteria

**Fig. 5:**
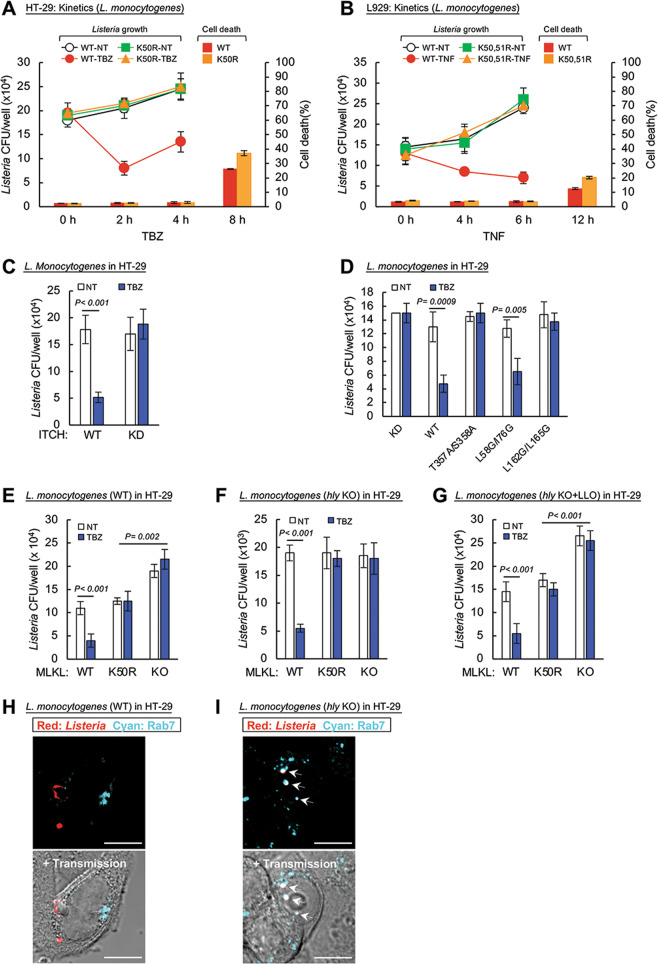
MLKL ubiquitination facilitates the destruction of *Listeria* in a manner distinct from the previously described direct cytotoxic effect of MLKL on these bacteria. **A**, **B** Kinetic analysis of the effects of TBZ treatment of HT-29 cells expressing the wild-type and the K50R mutant MLKL (**A**), and the effects of TNF treatment of L929 cells expressing wild-type and K50,51R mutant MLKL (**B**), on the amounts of viable *Listeria* in the cells and on the extent of cell death. As described in Supplementary Fig. 19, TNF was applied to HT-29 cells 3 h after initiation of infection, and to L929 cells 2 h after initiation of infection. Timings of bacterial yield quantification presented in the figure, therefore, correspond to 3, 5, and 7 h of infection period in **A**, and 2, 6, and 8 h in **B**. At the multiplicity of infection employed in this study (20 in HT-29 cells and 15 in L929 cells) *Listeria* had no effect on the extent of cell death. Expression of identical amounts of wild-type and mutant MLKL in the tested cells was confirmed by western blot analysis (Supplementary Fig. 16). **C** Effect of ITCH knockdown on the amount of viable *Listeria* in infected HT-29 cells 5 h after infection, and on its modulation by TBZ treatment. **D** Effects of various MLKL mutants constitutively expressed in MLKL knocked-down HT-29 cells, and of treatment with TBZ, on the amounts of viable *Listeria* in the cells. **E**–**G** Comparison of the impacts of TBZ treatment, the K50 mutation in MLKL, and the KO of MLKL on the amounts of viable *Listeria* in HT-29 cells, 5 h after infection with **E** wild-type *L. monocytogenes*, **F** the LLO mutant strain of *L. monocytogenes* DP-L2161, and **G** the latter strain in which LLO expression was reconstituted by transformation with LLO-expressing cDNA. Unless otherwise stated, in this and in all other presented experiments the durations of cellular infection by the indicated bacteria and the timing and duration of treatment with TBZ or with TNF were as specified in Supplementary Fig. 19. Each of the experiments presented in panels **A**−**G** was carried out three times, with duplicates of the samples (*n* = 6). **H**, **I** Immunocytochemical analysis of the intracellular location of *Listeria* compared to that of the endosomal marker Rab7, at 5 h after infection of HT-29 cells with the wild-type bacteria (**H**) or with the DP-L2161 strain (**I**). Arrows point to *Listeria* colocalized with Rab7 (as determined by staining of the two with their specific antibodies). In all experiments presented in this figure, MLKL and its mutants were re-expressed inducibly in MLKL KO cells.

As shown in Fig. 5A, B, in HT-29 and L929 cells expressing WT MLKL, induction of MLKL phosphorylation resulted in a substantial decrease in the rate of intracellular growth of *L. monocytogenes*. No such induced decrease was observed, however, in cells expressing the K50R human MLKL or the mouse K50,51R MLKL mutants, suggesting that this decrease occurs as a consequence of the ubiquitination of MLKL. Consistently, knockdown (KD) of ITCH—the enzyme that we found to mediate this ubiquitination—abolished the anti-listerial effect of TBZ (Fig. 5C).

This anti-listerial effect could be clearly distinguished from the cytotoxic effect of MLKL. The former occurred as early as 2 h after the induction of MLKL phosphorylation, well before any sign of cell death could be observed (Fig. 5A, B). Moreover, on comparing the effects of various MLKL mutants on the bacteria, we found on the one hand that MLKL mutants that cannot be phosphorylated (T357A/S358A), or do not oligomerize following phosphorylation (L162G/L165G), failed to mediate arrest of *Listeria* growth in response to TBZ. On the other hand, an MLKL mutant that is both phosphorylated and oligomerized in response to TBZ but fails to mediate necroptosis (L58G/I76G [8]) did mediate arrest of *Listeria* growth in response to TBZ (Fig. 5D).

A recent study suggested that MLKL can also suppress the growth of *L. monocytogenes* independently of MLKL oligomerization, upon its direct binding to the bacteria [36]. We found that mere KO of MLKL in the HT-29 cells consistently resulted in increased bacterial yield (Fig. 5E), despite the fact that—unlike after treatment with TBZ—the bacteria did not induce oligomerization or ubiquitination of MLKL (Supplementary Fig. 17).

Previous studies have shown that the extent to which *Listeria* keep on replicating in infected cells or are destroyed in them is subject to modulation by antagonistic mechanisms employed by this pathogen as well as by the host cells (see, e.g., [37–40]). The bacteria enter cells by co-opting receptor-uptake mechanisms. They are initially found within membrane-encased niches in the cells and then, with the assistance of a cholesterol-dependent cytolysin called “listeriolysin O” (LLO) that the bacteria produce, most of them escape to the cytoplasm [41]. Comparing the effect of MLKL on the growth of mutant *Listeria* that are deficient in LLO—and therefore cannot exit to the cytoplasm—with its effect on the WT bacteria and on the mutated bacteria in which LLO was re-expressed, we found that a decrease in bacterial yield that was due to mere expression of MLKL could be observed only in cells infected with the LLO-expressing bacteria. In contrast, the decrease imposed by induction of MLKL phosphorylation occurred independently of bacterial LLO expression (Fig. 5E−G). Immunostaining of the infected cells confirmed that most of the WT bacteria were found in the cytoplasm, whereas the mutant LLO-deficient bacteria occurred almost entirely in association with the endosomes (Fig. 5H, I).

These findings suggested that MLKL has two distinct effects on *Listeria*, which are exerted differentially at distinct sites in the cell: a direct toxic effect that MLKL exerts on the bacteria upon encountering them in the cytoplasm [36], and an effect of MLKL on those bacteria that have remained within membrane-encased niches which is exerted as a consequence of the binding of ubiquitinated MLKL molecules to endosomal membranes.

### MLKL ubiquitination also facilitates the destruction of some other intracellular bacteria

**Fig. 6:**
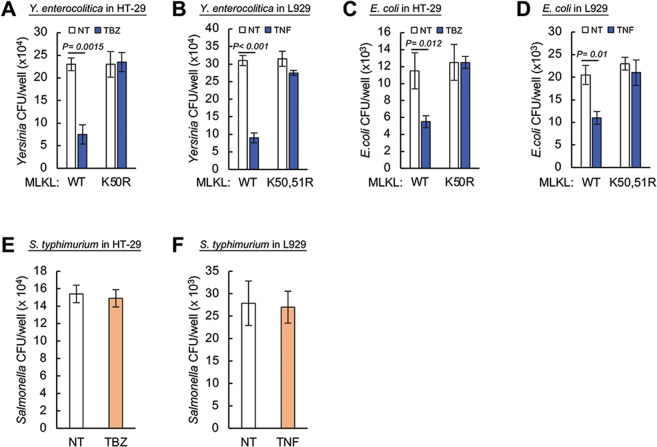
MLKL ubiquitination also facilitates the destruction of some other intracellular bacteria. **A**−**C** Yields of the indicated bacteria 5 h after infection of HT-29 cells or their HT-29 K50R mutant, and 6 h after infection of L929 cells or their K50,51R mutant, and the modulation of these yields obtained by treatment of the HT-29 cells with TBZ and treatment of the L929 cells with TNF. **D** Yields of *E. coli* 8 h after infection of L929 cells or their K50,51R mutant, and the modulation of these yields obtained by treatment of the L929 cells with TNF. **E** Yield of *S. typhimurium* 5 h after infection of HT-29 cells, and modulation of the yield by TBZ treatment. In **A** and **B**, three independent tests were performed (*n* = 6). **C** and **D** were done twice (*n* = 4). **F** Yield of *S. typhimurium* 8 h after infection of L929 cells, and modulation of the yield by TNF treatment. **E** and **F** were performed twice (*n* = 4). In all experiments presented in this figure, MLKL and its mutants were re-expressed inducibly in MLKL KO cells.

Similarly to *L. monocytogenes*, the Gram-negative bacterium *Yersinia enterocolitica* enters cells by co-opting receptor-uptake mechanisms, and its replication within the infected cells is subject to modulation by antagonistic effects exerted both by the bacteria and by cellular defense mechanisms [42, 43]. On assessing the yields of these bacteria in infected HT-29 and L929 cells we found that, as with *Listeria*, in cells that express WT MLKL the yield of *Y. enterocolitica* was effectively decreased by their treatment with TBZ and TNF, respectively, whereas in HT-29 cells expressing the K50R MLKL mutant and in L929 cells expressing the K50,51R MLKL mutant, these treatments had no effect on their bacterial yields (Fig. 6A, B).

We also examined the impact of MLKL activation on the infection of these cells by *Escherichia coli*, a bacterium that mainly grows extracellularly but can also adhere to mammalian cells and be phagocytized by them. We found that the yields of *E. coli* that could be recovered from HT-29 and L929 cells were decreased by TBZ and TNF treatments, similarly to the effects exerted by those respective treatments on the yields of *L. monocytogenes* and *Y. enterocolitica*, and that these effects were also compromised by mutation of lysine 50 in human MLKL or of lysines 50 and 51 in mouse MLKL (Fig. 6C, D). No such effects of TBZ and TNF were observed, however, on the retrospective yields of *Salmonella typhimurium* in the HT-29 and L929 cells (Fig. 6E, F).

### The anti-bacterial effect of ubiquitinated MLKL is mediated by the enhancement of bacterial translocation in the endosomes towards the lysosomes

**Fig. 7:**
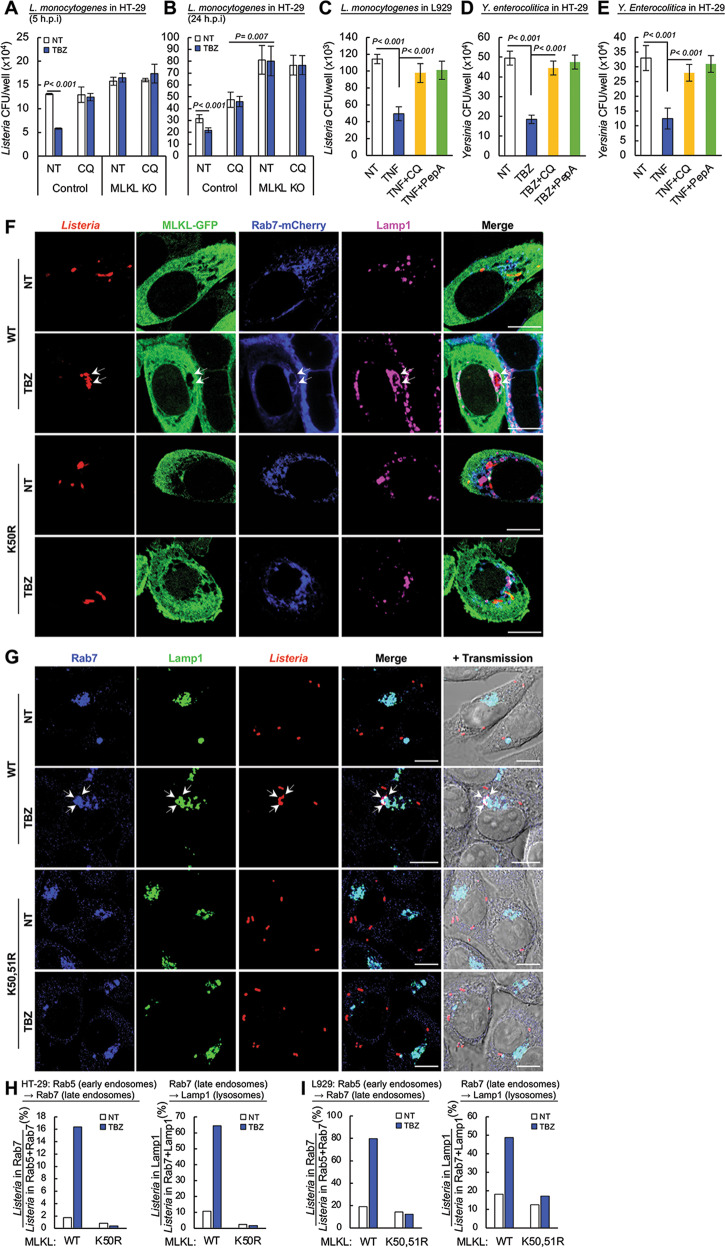
The anti-bacterial effect of ubiquitinated MLKL is mediated by enhancing the translocation of the bacteria from the endosomes to the lysosomes. **A**, **B** Effects of chloroquine (CQ, 25 μM), applied throughout the times of infection of HT-29 cells by *Listeria* on the bacterial yield and on its modulation by MLKL. Comparison of the amounts of viable *Listeria* at 5 h (**A**) and at 24 h (**B**) post infection (h.p.i), and of the effects of TBZ treatment for 2 h at the end of the infection period, in MLKL KO HT-29 cells and in KO cells that inducibly express either wild-type MLKL or its K50R mutant. **C** Effect of chloroquine (CQ, 25 μM) and of pepstatin A (PepA, 10 μg/ml), applied to L929 cells as in **A** and **B**, on the TNF-induced reduction in *Listeria* yields. TNF was applied to the cells for 6 or 8 h following their infection with *Listeria*. **D** Effects of chloroquine and of pepstatin A, applied to HT-29 cells as in **A** and **B**, on the TBZ-induced reduction in *Y. enterocolitica* yields. **E** Effects of chloroquine and of pepstatin A, applied for 4 h to L929 cells together with TNF, at 6 h after infection of the cells with *Y. enterocolitica*, on the TNF-induced reduction of the bacterial yield in these cells. In **A**−**E**, three independent tests were performed (*n* = 6). **F**, **H** Immunocytological analysis of the location of *Listeria*, 5 h after infection, in early and late endosomes, in lysosomes and in the cytosol, and its TBZ-induced modulation, in MLKL KO HT-29 cells inducibly expressing wild-type and K50R-mutated MLKL fused C-terminally to GFP. **F** Typical immunofluorescence images. Arrows point to *Listeria* colocalized with MLKL-GFP + Rab7 fused to mCherry + Lamp1. **H** Ratios of the numbers of *Listeria* in the indicated compartments (analysis of 130 infected cells). **G**, **I** Immunocytological analysis of the location of *Listeria* in early and late endosomes, in lysosomes and in the cytosol, 6 h after infection, and their TNF-induced modulation, in MLKL KO L929 cells inducibly expressing wild-type and K50,51R-mutated MLKL. **G** Typical immunofluorescence images. Arrows point to *Listeria* colocalized with Rab7 + Lamp1. **I** Ratios of the numbers of *Listeria* in the indicated compartments (analysis of 120 infected cells).

We have previously shown that MLKL downregulates the cellular response to epidermal growth factor (EGF) by enhancing the lysosomal destruction of this cell-growth factor and its receptor, and that MLKL similarly affects TNF following receptor-mediated uptake of this cytokine into the cell [23]. Those findings raised the possibility that the anti-bacterial effect of ubiquitinated MLKL is also mediated by the enhancement of lysosome-mediated destruction. Indeed, as shown in Fig. 7A, B, blocking of lysosomal function in HT-29 cells by means of chloroquine application obliterated the inhibitory effect of TBZ on *L. monocytogenes* growth. In the absence of TBZ the chloroquine had no effect on bacterial yield, nor did it affect the difference between bacterial yields in the MLKL KO and the WT cells. Chloroquine similarly affected the inhibitory effect of TNF on the growth of *L. monocytogenes* in mouse L929 cells (Fig. 7C). It also blocked the inhibitory effects exerted by TBZ and TNF on the growth of *Y. enterocolitica* in HT-29 and L929 cells, respectively (Fig. 7D, E). Pepstatin A, a cathepsin D inhibitor, also obliterated the inhibitory effects of TBZ and TNF on bacterial growth in HT-29 and L929 cells (Fig. 7C−E).

Both *Yersinia* and *Listeria* have been shown to activate autophagic mechanisms, and it was suggested that those mechanisms facilitate the destruction of the bacteria [44, 45]. Activation of MLKL was also reported to facilitate autophagy [46]. It, therefore, seems plausible that the lysosomal destruction of intracellular bacteria that we found to be induced by ubiquitinated MLKL is exerted via enhancement of autophagy. However, KO of ATG5—a protein that plays a crucial role in both canonical and non-canonical autophagy—had no effect on the extent of MLKL ubiquitination in TBZ-treated HT-29 cells or on the TBZ-induced decrease in the growth of *L. monocytogenes* or *Y. enterocolitica* (Supplementary Fig. 18). This rendered it unlikely that the effect of ubiquitinated MLKL on these bacteria reflects enhanced autophagy.

We have previously shown that MLKL enhances translocation of the EGF receptor in EGF-treated cells from the early to the late endosomes, and from there to the lysosomes [23]. By applying immunostaining to track *Listeria* in infected HT-29 cells along the endosomal pathway, we found that stimulation of MLKL phosphorylation and ubiquitination similarly enhances *Listeria* translocation from the early to the late endosomes and from there to the lysosomes, and that no such enhancement occurs in cells expressing the K50R MLKL mutant (Fig. 7F, H). Immunostaining of *Listeria*-infected L929 cells revealed similar increases in translocation of *Listeria* from the early to the late endosomes and from there to the lysosomes in response to TNF, which did not however occur in cells expressing the K50,51R-mutated mouse MLKL (Fig. 7G, I).

These findings suggested that the suppression of bacterial growth by stimulation of MLKL phosphorylation is an outcome of the enhancement of endosomal trafficking by ubiquitinated MLKL molecules.

## Discussion

A lesson repeatedly learnt since the advent of the cell-death field of investigation is that molecules that initially were identified by their contribution to programmed cell death actually also serve various non-deadly functions. In the case of those proteins that mediate cell death as a means of immune defense, this multiplicity of functions allows them to contribute to intricate sets of defense mechanisms in which death of the afflicted cell is just one of several defense-mode options. This lesson was initially learnt about proximal molecules in the signaling pathways that mediate death. Thus, though at first dubbed “tumor necrosis factor,” “death domain,” RIP (meaning “Rest in Peace”) and other death-associated names, these initiating molecules and structural motifs have, one after another, all eventually turned out to serve rather as pleiotropic regulators of defense. Subsequent studies have clarified that this is the case not only with the molecules and motifs that initiate signaling for death, but also with the most distal “effector molecules” in the death-signaling pathways. It is true, for example, for the caspases, which serve as the effector molecules in the signaling for apoptotic cell death, yet are now known also to serve numerous important non-deadly functions [47]. It is also now gradually being found to be true for effector molecules in the various forms of programmed necrotic cell death. The present study expands our knowledge of non-deadly functions of MLKL—the effector molecule in necroptotic death—and of the molecular determinants affecting the kinds of functions that MLKL serves in different situations.

In this study, we confirmed our earlier finding that some MLKL molecules associate with endosomal membranes and that this association is enhanced upon phosphorylation of MLKL by RIPK3 [23]. Unlike the association of MLKL with the cell membrane, which also occurs as a consequence of its phosphorylation by RIPK3, the association of MLKL with endosomal membranes does not trigger a death process, but quite to the contrary, since those MLKL molecules that associate with endosomes are eventually released from the cell within EVs, cells in which this association is extensive display increased resistance to inducers of necroptotic death [23]. In that earlier study, we showed that the association of MLKL with endosomes prompts enhanced translocation of certain cell-surface receptors and their ligands to the lysosomes [23]. In the present study, we show that the association of MLKL with endosomes also prompts enhanced endosomal transport of some intracellular pathogens.

The fact that MLKL and the proteins that activate it serve to facilitate immune defense against pathogens is indicated by emerging evidence that a variety of different pathogens have evolved mechanisms that act to antagonize functions of MLKL [48]. Thus, for example, several poxviruses encode homologs to the pseudokinase domain of MLKL that block MLKL activation by sequestering RIPK3 [49]. Also, a protease encoded by *Shigella* blocks such activation by degrading RIPK1 and RIPK3 [50].

Our knowledge of the mode of activation of MLKL, and of the kinds of effects that it exerts on cells, is also consistent with the notion that this protein is destined to assist the immune defense. This is indicated by the nature of its three known upstream regulators: RIPK1, a protein that acts downstream of immunomodulatory cytokines such as TNF; TRIF, which acts downstream of receptors for pathogen-associated molecular patterns and damage-associated molecular patterns, such as TLR4, and DAI/ZBP1, a receptor for Z-nucleic acid [6]. Moreover, NETosis—the expulsion of DNA by neutrophils in association with histones and cytoplasmic proteases, thereby trapping pathogens and killing them—was shown to be induced by some pathogenic agents (but not by others) in a way that depends on activation of the necroptotic signaling pathway [51–53]. Initial evidence has been presented for activation (downstream of the phosphorylation of MLKL) of certain molecular mechanisms that specifically contribute to this process (generation of reactive-oxygen species independently of activation of NADPH oxidase, and generation of hypercitrullinated histones via activation of peptidylarginine deiminase 4) [54]. Moreover, MLKL has been reported to mediate destruction of intracellular pathogens, and to do so in two ways previously described for anti-pathogenic effects of several other death-mediating molecules: killing of the host cells (thereby eliminating the pathogen’s niche), and selective killing of the pathogen itself as an outcome of MLKL binding to it. Thus, growth of *Leishmania infantum* in neutrophils was found to be blocked by necroptotic death of the neutrophils [55], while the growth of *L. monocytogenes* in epithelial cells was found to be blocked by MLKL through a direct toxic effect of the protein on the pathogen’s cells [36]. In the present study, we report a third anti-pathogenic role of MLKL, which differs from the above two both mechanistically and in the nature of its consequences. Our findings suggest that MLKL, once ubiquitinated, acquires the ability to initiate anti-bacterial effects that are mediated, not via its direct interaction with the bacteria nor by the killing of the pathogen’s host cell, but rather by targeting of the bacteria to lysosomes.

Mutation of lysine 50 in human MLKL, or of both lysines 50 and 51 in mouse MLKL, is shown in this study to ablate the enhancement of bacterial targeting to lysosomes by phosphorylated MLKL. We also found that mutation of the same lysines ablates the MLKL-induced enhancement of the endosomal trafficking of EGF and its receptor (data not shown). Lysine 50 in human MLKL and lysines 50 and 51 in mouse MLKL are shown here to be major sites of the K63-linked ubiquitination of MLKL following its phosphorylation and oligomerization. This ubiquitination is mediated by the E3 ligase ITCH, which associates inducibly with MLKL upon induction of MLKL phosphorylation. A causal relationship between this ubiquitination and the effects of MLKL on endosomes is indicated by the observed association of ubiquitinated MLKL with endosomes. It is also indicated by the preferential release of ubiquitinated MLKL in vesicles that seem to emerge from the endosome-derived multivesicular bodies, and by the vast decrease in MLKL release within these vesicles when the ubiquitination sites are mutated. In view of prior evidence for ubiquitin recognition by ESCRT proteins [56], it seems possible that the recruitment of ubiquitinated MLKL to endosomes is dictated by recognition of the ubiquitin chains by certain ESCRT proteins. However, the mechanism by which such recruitment prompts enhanced endosomal flux is unknown.

During the time of preparation of this paper, two other studies about the ubiquitination of MLKL and its functional roles were published ([28] and [57]). Both studies confirmed that MLKL is ubiquitinated following its RIPK3-mediated phosphorylation. However, under the particular testing systems employed in those two studies, the ubiquitination of MLKL that occurs prior to cell death could not be examined. (In fact, in both studies it was stated that MLKL ubiquitination could be discerned only at the time of cell death.) Therefore, although—similarly to our study—in both of those studies it was reported that mouse MLKL is ubiquitinated in lysine K51, and in one it was pointed out that the MLKL molecules ubiquitinated at that site are associated with a particulate cellular subfraction, in both studies the focus was on ubiquitination of other lysine residues. The functional roles of the ubiquitination suggested in those two studies differ from the one we describe here. In one of the two, it was suggested that MLKL ubiquitination serves a crucial role in triggering cell death by assisting the oligomerization of MLKL molecules [28], while in the other the MLKL ubiquitination suggested rather to withhold cell death by dictating proteasomal and lysosomal degradation of MLKL [57].

Our finding that the protein which mediates necroptotic cell death dictates, prior to such mediation, intracellular destruction of pathogens, seems to make physiological sense in view of the particular features of programmed necrosis. In apoptotic cell death, the dying cell is already taken up by phagocytic cells before its cell membrane has ruptured, allowing the destruction of any still-living pathogens by mechanisms executed by the phagocytic cell engulfing the apoptotic cell. Necrotic death, on the other hand, is characterized by rupture of the cell membrane at the time of death. Enhancement of the lysosomal targeting of pathogens soon after necroptosis induction but before the actual occurrence of cell death can thus serve as a safeguard against the release of live pathogens from the dying cell. Assessment of the impact of mutation of the ubiquitination site in MLKL in experimental animal models should enable us to obtain concrete knowledge of the relative contribution of this mechanism to the defense against specific pathogens, and might also reveal additional functions served by MLKL upon its binding to endosomes.

## Materials and methods

### Mice

MEFs were derived from WT and *Mlkl*^*−/−*^ mice on C57BL/6J background. The *Mlkl*^*−/−*^ mice were obtained from Taconic. The protocol for fibroblast generation was approved by the Institutional Animal Care and Use Committee of The Weizmann Institute of Science.

### Cell culture

Cells of the human HT-29 colorectal adenocarcinoma line were grown in McCoy’s 5A medium. Normal MEFs and embryonic fibroblasts of MLKL KO mice were both immortalized by the expression of the SV40 large T antigen. Immortalized MEFs, cells of the mouse L929 fibroblast line, and human embryonic kidney HEK293T cells were cultured in Dulbecco’s modified Eagle’s medium. The cell-growth media were supplemented with 10% fetal bovine serum, 100 U/ml penicillin, and 100 μg/ml streptomycin.

### Reagents

Bafilomycin A1 (11038-500) and pepstatin A (9000469-10) from Cayman Chemical were applied to the cells at 100 nM and 10 μg/ml respectively. Chloroquine (C6628), MG132 (M7449), and 4-hydroxytamoxifen (4HT, H6278) from Sigma-Aldrich were applied at 25, 10, and 1 µM, respectively. To trigger necroptosis in HT-29 cells, human TNF from YbdY Biotech (1000 units/ml) was applied together with BV6 [58] and z-VAD-fmk, both from WuXi AppTec, at concentrations of 1 and 20 µM, respectively. Necroptosis was induced in the L929 cells by treatment with TNF (1000 U/ml) alone.

Strep-Tactin XT Superflow, 50% suspension (2-4010-025), and Strep-Tactin XT Elution Buffer (2-1042-025) were from IBA. Agarose-coupled TUBE1 (UM401) and K63-TUBE (UM604) were from LifeSensors. NI-NTA His-BIND resin (70666) was from Novagen. Brij O10 (polyoxyethylene 10 oleyl ether, P6136) and *N*-ethyl-maleimide (E3876) were from Sigma-Aldrich. The Lipofectamine 3000 transfection reagent (L3000015), CellLight Early Endosomes-green fluorescent protein (GFP) (10586), and CellLight ER-GFP (C10590) were from Thermo Fisher Scientific, and the JetPEI transfection reagent (101-10N) was from Polyplus-transfection. 1,10 Phenanthroline (SI 9649), ubiquitin-aldehyde (SI 250), and PR-619 (SI 9619) were from LifeSensors. Mouse interferon-β (12401-1) was from PBL Assay Science. Poly I:C (tirl-picw) was from Invivogen. Recombinant human MLKL (CSB-EP850851HU) and mouse MLKL (CSB-EP861529MO) were from Cusabio Technology and recombinant ITCH (E3-260) was from R&D Systems.

### Antibodies

The following antibodies were used for western blot analysis: anti-mouse MLKL (Sab1302339, 1:500), anti-HA (H6908, 1:1000), anti-β-actin (A5441, 1:10000), anti-Flag M2 (F3165, 1:2500), and anti-MVB12B (HPA043683, 1:200) from Sigma; anti-human MLKL (ab184718, 1:2000), anti-human phospho MLKL (ab187091, 1:500), anti-mouse phospho MLKL (ab196436, 1:1000), anti-human phospho RIP3 (ab209384, 1:2000), anti-human Rab7 (ab 137029, 1:1000), anti-K63-specific ubiquitin (ab179434, 1:1000) and VeriBlot for IP detection reagent (ab131366, 1:4000) from Abcam; anti-TSG101 (612696, 1:1000), anti-ITCH (611199, 1:1000) and anti-EEA1 (610456, 1:5000) from BD Biosciences; anti-LDH (sc-27230, 1:500), anti-STIM-1 (sc-166840, 1:500) and anti-TOM20 (sc-17764, 1:500) from Santa Cruz; anti-ubiquitin (VU101, 1:1000) from LifeSensors; anti-myc tag (05-724, 1:1000) from Millipore; anti-HSP70 (EXOAB-hsp70A-1, 1:1000) from System Biosciences; anti-human RIP3 (VMA00393, 1:1000) from Bio-Rad, anti-Strep-tag (34850, 1:2000) from Qiagen; anti-Na^+^/K^+^ ATPase (ANP-001, 1:500) from Alomone Labs, anti-VPS28 (NBP1-85976, 1:100) from Novus Biologicals, and horseradish peroxidase-conjugated antibodies (1:10000) from Jackson ImmunoResearch.

The following antibodies were used for immunostaining: Alexa Fluor 647-conjugated anti-mouse IgG (Jackson ImmunoResearch, 715-605-150, 1:300), anti-ubiquitin (FK2, Enzo Life Sciences, BML-PW8810-0500, 1:200), anti-ITCH (BD Biosciences, 611199, 1:100), anti-mouse Lamp1 (Developmental Studies Hybridoma Bank, 1D4B, 1:200). Anti-human Lamp1 (ab25630, 1:200), anti-human MLKL (ab184718, 1:200), anti-human Rab7 (ab 137029, 1:200), anti-mouse Rab7 (ab50533, 1:200) from Abcam, anti-mouse Rab5 (R7904, 1:200) from Sigma. Alexa Fluor 405-conjugated anti-mouse IgG (ab175660, 1:300), and anti-*Listeria* (ab35132, 1:400) were from Abcam. The fidelity of the anti-human MLKL antibody was confirmed by comparing the immunostaining of WT and MLKL KO HT-29 cells.

### Immunocytological analyses by fluorescence microscopy

For immunocytological analyses, cells were fixed with 4% paraformaldehyde for 15 min at room temperature in phosphate-buffered saline (PBS), permeabilized with ice-cold methanol for 10 min at −20 °C, and blocked with MAXblock Blocking Medium (Active Motif, 15252) overnight at 4 °C. The cells were then incubated with the indicated antibodies for 1 h at room temperature, and then with fluorescent dye-conjugated secondary antibodies for 2 h at 4 °C in MAXbind Staining Medium (Active Motif, 15253). Images were acquired with an Olympus IX 81 confocal microscope (Olympus Imaging) using the UPLSAPO 60× /1.35 NA oil objective, and were processed using the FluoView FV1000 (Olympus Imaging) and ImageJ (NIH) software.

Unless otherwise stated, early and late endosomes in the HT-29 cells were visualized by constitutive expression of either Rab5 or Rab7 in fusion with mCherry. Exceptions to that were the experiments presented in Fig. 5H, I and Supplementary Fig. 10, in which late endosomes were visualized using an anti-Rab7 antibody (ab 137029 from Abcam), and those presented in Supplementary Fig. 9, in which the early endosomes were visualized using the CellLight Early Endosomes kit. Early and late endosomes in the L929 cells were visualized by immunostaining of Rab5 or Rab7 using specific antibodies (anti-mouse Rab5 (R7904) from Sigma and Rab7 (ab50533) from Abcam).

MLKL was visualized in the experiment presented in Supplementary Fig. 9 by immunostaining with a specific antibody (anti-MLKL (ab184718) from Abcam) and in all other experiments by inducible expression of MLKL fused C-terminally to GFP.

### Bimolecular fluorescence complementation (BiFC) analysis

Colocalization of ubiquitin and MLKL in HT-29 cells by BiFC was assessed as described [59]. MLKL, fused C-terminally to a yellow fluorescent protein (YFP; 173−251), was expressed by retroviral infection in the pBabe puro plasmid (Addgene, 1764). Ubiquitin, fused N-terminally to YFP (1−172), was expressed by lentiviral infection using the pLV-EF1a-IRES-Hygro vector (Addgene, 85134). The location of the ubiquitinated MLKL was determined by confocal microscopy as described above. In these experiments, endosomes were visualized by immunostaining of Rab7.

### Gene knockout (KO) and knockdown (KD) in vitro in human HT-29 and mouse L929 cells

In the human HT-29 cells, MLKL was knocked out in vitro using the MLKL CRISPR/Cas9 KO plasmid (Santa Cruz, sc-401248) transfected with Lipofectamine 3000. FADD was knocked out by transient transfection with two gRNA sequences (5’-GTTCCTATGCCTCGGGCGCGT, 5’-ACGCGCCCGAGGCATAGGAAC) in the pX459 plasmid (Addgene, 62988). ITCH was knocked out in HT-29 cells by lentiviral infection with two gRNA sequences (5’-GGTGCTTCTCAGAATGATGA, 5’-TGAACATGTAGTTTCACCAT) and a single gRNA sequence (5’-CGAAAACATAATTTACTCGT) in mouse cells, using the pLenti CRISPR V2 vector (Addgene, 52961). Control cells were infected with the pLenti CRISPR V2 vector expressing a non-targeting scramble sequence (5’-ACGGAGGCTAAGCGTCGCAA). NEDD4 was knocked out by lentiviral infection with the 5’-TTCGGAAATGGCAACTTGCG and 5’-CCAACCGGTAATGGATAAAG gRNA sequences, ATG5 with the 5’-AAATGTACTGTGATGTTCCA and 5’-AAGAGTAAGTTATTTGACGT gRNA sequences, and mouse MLKL with the 5’-CCCAACATCTTGCGTATATT and 5’-AGGAACATCTTGGACCTCCG gRNA sequences, all in the pLenti CRISPR V2 Neo vector. KO of human MLKL in HT-29 cells and of mouse MLKL in L929 cells was validated by western blot analysis using MLKL-specific antibodies, ab184718 from Abcam and Sab1302339 from Sigma, respectively. KO and KD of other genes were validated by western blotting using the specific antibodies listed in the “Antibodies” section above.

For KD of ITCH, SMARTpool On-TARGETplus human ITCH siRNA (L-007196-00-0050, Dharmacon) was transiently transfected into HT-29 cells, and the cells were further incubated for 3 days.

### Constitutive and inducible expression of proteins in cultured mammalian cells

Unless otherwise indicated, MLKL and its various mutants that were re-expressed in MLKL KO and MLKL KD cells were fused C-terminally to a double Strep-tag (**WSHPQFEK**-GGGSGGGSGGGS-**WSHPQFEK**). Human MLKL T357A/S358A and L162G/165G mutants (and, as a control, the WT protein) were expressed constitutively in MLKL knocked-down cells as previously described [20].

Site-directed mutations were introduced in human MLKL cDNA (Origene, RC213152) and mouse MLKL cDNA (Origene, MR207406) using the QuickChange II Site Directed Mutagenesis kit (Agilent, 200521). The GEV16/pF5x UAS system [60] was used to inducibly express, in MLKL KO cells, the various human and mouse MLKL mutants in which lysine residues were replaced with arginine (and, as controls, the WT proteins) and human MLKL fused C-terminally to GFP. In the experiment presented in Fig. 4J, this system was also used for the expression of ITCH that was fused C-terminally to myc tag (EQKLISEEDL). Unless otherwise indicated, induction was by treatment with 4HT (1 μM) for 6 h. In the experiment presented in Fig. 4F, the various ITCH deletion mutants, which were fused C-terminally to the FLAG-tag (DYKDDDDK), were expressed by transient transfection in HEK293T cells using JetPEI. HA-ubiquitin and its K63R mutant, and Rab5 and Rab7 fused N-terminally to mCherry, were expressed constitutively by lentiviral infection.

### Collection of extracellular vesicles

EVs generated by HT-29 cells were isolated as previously described [23].

### Isolation of subcellular fractions

For the experiment presented in Fig. 3A, subconfluent cultures of HT-29 cells in eight 150-mm plates were incubated for 3 h with TBZ. After the cells were rinsed twice with PBS they were collected by scraping, and homogenized in a buffer containing 225 mM mannitol, 75 mM sucrose, 0.5 mM EGTA, and 30 mM Tris-HCl, pH 7.4, using a loose-fitting glass/Teflon Potter-Elvehjem homogenizer followed by repeated passage through a 22-gauge syringe. Fractionation by differential centrifugation was performed as previously described [61].

For the experiment presented in Supplementary Fig. 8, endosomal membranes were isolated by a discontinuous sucrose gradient method [62].

### Immunoprecipitation and western blot analysis

Protease and phosphatase inhibitors (1 mM phenylmethylsulfonyl fluoride (PMSF), 40 mM beta-glycerophosphate, 50 mM sodium fluoride (NaF), and 1 mM sodium orthovanadate (NaV)) were included with all the extraction buffers that were used for immunoprecipitation. Unless otherwise indicated, proteins were immunoprecipitated following the extraction of cells with 1% NP-40 lysis buffer (50 mM Tris-HCl pH 7.5, 150 mM NaCl, 1 mM EDTA, 1% NP-40) as described [23]. To estimate the extent of oligomerization of MLKL, the extraction was followed by dilution of the extracts with Laemmli sodium dodecyl sulfate−polyacrylamide gel electrophoresis (SDS–PAGE) buffer devoid of any reducing agent, as described [8]. The protein interactions with MLKL that are presented in Fig. 4J were assessed using HT-29 KO cells that inducibly express MLKL fused C-terminally to the double Strep-tag [63]. In this experiment, the cells were extracted in a buffer containing 1% Brij, as described [23]. In the experiments presented in Fig. 4C, and in Supplementary Figs. 3, 12, and 13, the protein interactions were assessed after extraction of the cells with RIPA buffer (20 mM Tris-HCl pH 7.5, 150 mM NaCl, 1 mM EDTA, 1% NP-40, 0.5% deoxycholate, 0.1% SDS).

To detect K63-linked ubiqitination using K63-specific antibody (Fig. 2D), strep-tag-fused MLKL or its K50R mutant was expressed inducibly in MLKL KO HT-29 cells. Cell samples (1.2 × 10^8^ cells) were treated with TBZ for 3 h, then rinsed with ice-cold PBS and lysed with 1% NP-40 lysis buffer. After preclearing of the protein extract with agarose beads, MLKL was precipitated using Strep-Tactin XT beads, followed by its elution using Strep-Tactin XT elution buffer. The eluted protein samples were analyzed by SDS–PAGE. The blot was developed using SuperSignal West Femto Maximum Sensitivity Substrate (34096, Thermo Fisher Scientific).

### Quantification of cell death

Cell death was quantified by determining the concentration of lactic dehydrogenase in cell-culture media, using the Cytotoxicity Detection Kit of Sigma-Aldrich (4744934001).

### Assessment of MLKL ubiquitination

Samples of 6 × 10^6^ HT-29 cells, 8 × 10^6^ MEFs, and 12 × 10^6^ L929 cells, seeded 1 day prior to the experiment, were treated with 1 µM 4HT for 12 h to induce expression of WT or mutant MLKL. Comprehensive isolation of all forms of ubiquitinated MLKL from cell extracts using TUBE1-agarose, and (using K63-TUBE) isolation of only those MLKL molecules to which K63-linked polyubiquitin chains are conjugated, were performed according to the manufacturer’s protocol, except that the extraction buffer also contained protease and phosphatase inhibitors (1 mM PMSF, 40 mM beta-glycerophosphate, 50 mM NaF, and 1 mM NaV).

To assess the incorporation of HA-tagged ubiquitin chains to MLKL, WT and mutant HA-tagged ubiquitins were expressed constitutively by lentiviral infection in cells inducibly expressing MLKL fused to Strep-tag, and—following growth of the cells for a further 12 h and, when indicated, stimulation by TBZ—the cells were lysed by boiling them in a buffer containing 1% SDS, 20 mM Tris-HCl (pH 7.5), 5 mM EDTA, and 1 mM dithiothreitol (DTT), followed by ten-fold dilution of the lysates in a buffer containing 1% NP-40, 20 mM Tris-HCl (pH 7.5), 50 mM NaCl, 5 mM EDTA and the protease and phosphatase inhibitors specified above. MLKL was affinity-purified using Strep-Tactin beads, and the extent of conjugation of its tagged ubiquitin chains was assessed by western blotting using anti-HA antibody.

### In vitro ubiquitination and deubiquitination of MLKL

All enzymes and recombinant proteins were from R&D Systems. For the in vitro MLKL ubiquitination assay, WT or K50R MLKL, affinity-purified using Strep-Tactin beads following denaturation in SDS and further treatment with NP-40-containing buffer as described above, were incubated for 1 h at 37 °C with recombinant E1 (UBE1, E-305; 50 nM), E2 (UBE2L3, E2-640; 200 nM), and E3 (ITCH, E3-260; 1 μM) enzymes, ubiquitin (U-100H, 80 μM), Mg-ATP (B-20, 10 mM), and 10× E3 ligase reaction buffer (B-71).

For the in vitro deubiquitination assay, MLKL, which was affinity precipitated using Strep-Tactin beads following its denaturation in SDS and further treatment with NP-40 as described above, was incubated with the indicated recombinant deubiquitinases (USP2(E-506), 200 nM; Otubain1 (E-522B), 1 μM; AMSH (E549), 1 μM or CYLD (E-556), 20 nM), for 1 h at 37 °C in a reaction buffer containing 50 mM Tris pH 7.5, 5 mM MgCl_2_, 25 mM KCl and 1 mM DTT. Synthetic K63-linked and K48-linked ubiquitin chains (UM-K630 and UM-K480, R&D Systems) served as controls.

### Bacterial infection and quantification

HT-29 cells and L929 cells, seeded 1 day before infection, were incubated, prior to being infected, for 10 h in serum-free BioGro-2 medium (Biological Industries, 05-610-1B, Israel) without antibiotics.

WT *L. monocytogenes* (strain 10403S, kindly provided by Dr Steffen Jung), the LLO mutant (*hly* knockout) strain of *L. monocytogenes* DP-L2161 (kindly provided by Dr Daniel A. Portnoy [64]), *Y. enterocolitica* (ATCC 27729, kindly provided by Dr Emmy Mamroud), *S. enterica* serovar Typhimurium-expressing GFP in pFPV25.1 plasmid (strain SL1344, kindly provided by Dr Roi Avraham), and *E. coli* DH5a (New England BioLabs, C2987H) transformed with a pcDNA 3.1 plasmid (Thermo Fisher Scientific, V79020) were applied to the cells in serum-free BioGro-2 medium, followed by centrifugation at 300 × g for 10 min and incubation at 37 °C for 1 h to allow invasion of the bacteria. The cells were then rinsed with PBS, incubated with 100 μg/ml gentamicin (Sigma-Aldrich, G1397) for 10 min and then with a fresh growth medium containing 5 μg/ml of gentamicin. Protocols of the infection and subsequent treatments of the cells are presented in Supplementary Fig. 19.

To quantify the intracellularly grown bacteria, infected cells were rinsed twice with PBS and then lysed in 0.2% Triton X-100 in PBS (1 ml per 22-mm well). Following serial dilution of the lysates, we quantified their contents of *L. monocytogenes* and *Y. enterocolitica* by spreading them on BHI agar plates, and of intracellular *S. enterica* and *E. coli* by spreading them on LB agar plates containing ampicillin (200 μg/ml).

### Reconstitution of Listeriolysin O (LLO) expression in the *hly* KO *Listeria* (DP-L2161)

LLO cDNA was cloned into the pPL2 plasmid and introduced into the *hly* KO *Listeria* strain DP-L2161 by conjugation with *E. coli* SM10. Following the selection of the bacteria on BHI agar plates with 50 μg/ml streptomycin (Sigma-Aldrich, S9137) and 15 μg/ml chloramphenicol (Sigma-Aldrich, C0378), they were grown overnight in the presence of 100 ng/ml anhydrous tetracycline (Sigma-Aldrich, 37919). The pPL2 plasmid and *E. coli* SM10 were kindly provided by Dr Anat Herskovits.

### Mass spectrometry-based proteomics analysis

#### MLKL purification

MLKL fused to Strep-Tag was expressed in HT-29 cells by their treatment with 1 μM 4HT for 6 h, and its phosphorylation was induced by treatment with TBZ for the last 3 h of the expression period. Cells were rinsed twice with ice-cold PBS and then lysed in 6 M urea buffer (100 mM Tris pH 8.0, 150 mM NaCl, 1 mM EDTA, 6 M urea) that included a protease inhibitor cocktail (Roche, 11836145001), phosphatase inhibitors (1 mM PMSF, 40 mM beta-glycerophosphate, 50 mM NaF, and 1 mM NaV) and ubiquitin isopeptidase inhibitors (4 mM 1,10 phenanthroline, 1 μM ubiquitin-aldehyde, 10 mM *N*-ethyl-maleimide, and 50 μM PR-619). MLKL was precipitated using Strep-Tactin XT beads after clearing of the protein extract with agarose beads, followed by its elution using Strep-Tactin XT elution buffer.

#### Sample preparation

Proteins were denatured with 2 M urea (final concentration), then reduced with 5 mM DTT (Sigma) for 1 h at room temperature and alkylated with 10 mM iodoacetamide (Sigma) in the dark for 45 min at room temperature. Samples were diluted in a buffer containing 2 M urea with 50 mM ammonium bicarbonate. Proteins were then subjected to digestion with 300 ng trypsin (Promega) overnight at 37 °C, followed by second trypsin digestion for 4 h. Digestion was stopped by the addition of trifluoroacetic acid (1% final concentration). Digested peptides were desalted using the Oasis HLB, μElution format (Waters). Samples were vacuum dried and stored at −80 °C until further analysis.

The resulting peptides were then enriched using the PTMScan Ubiquitin Remnant Motif (K-ε-GG) Kit (Cell Signaling Technology, 5562), according to the manufacturer’s instructions. For the targeted experiment, four synthetic heavy labeled peptides, purchased from JPT Peptide Technologies—F**K(GG)**AALEEANGEIEK, LHHSEAPELHG**K(GG)**IR, VLGLIKPLEMLQDQG**K(GG)**R, and VLGLIKPLE**M(Ox)**LQDQG**K(GG)**R—were added to the samples at 50, 50, 200, and 400 fmol/µL, respectively.

#### Liquid chromatography

ULC/MS-grade solvents were used for all chromatographic steps. The mobile phase was: (i) H_2_O + 0.1% formic acid and (ii) acetonitrile + 0.1% formic acid. Samples were desalted online using a reversed-phase Symmetry C18 trapping column (180 µm internal diameter, 20 mm length, 5 µm particle size; Waters). The peptides were then separated using a T3 HSS nano-column (75 µm internal diameter, 250 mm length, 1.8 µm particle size; Waters) at 0.35 µL/min. For the profiling experiment, each sample was loaded using the Ultimate 3000 liquid chromatography system (Thermo Fisher Scientific). For the targeted experiment we used the nanoACQUITY UPLC system (Waters).

#### Mass spectrometry

The nanoUPLC system was coupled online through a nanoESI emitter (10 μm tip; New Objective) to a quadrupole orbitrap mass spectrometer (Exploris 480 or Q Exactive HF, Thermo Fisher Scientific) using a FlexIon nanospray apparatus (Thermo Fisher Scientific).

For the profiling experiment, data were acquired by data-dependent acquisition. MS1 resolution was set at 120,000, automatic gain control (AGC) at 200%, maximum injection time at 100 ms, mass range at 380–1500 Th.

For the targeted experiment, data were acquired in both full MS1 scan and parallel reaction monitoring (PRM) mode. For MS1, the resolution was set at 120,000, AGC target at 1e6, injection time at 50 ms, and mass range at 375–1500 Th. For PRM, MS2 resolution was set at 30,000, AGC target at 5e5, and maximum injection time at 150 ms. Eight precursor masses were targeted, corresponding to the light and heavy masses of the four GG-modified peptides.

#### Data processing

For the profiling experiment, raw mass spectrometry data were processed using MetaMorpheus version 0.0.311 (https://pubs.acs.org/doi/abs/10.1021/acs.jproteome.6b00034) against the human database from UniproKB. Allowed modifications included GG on Lys, oxidation of Met, and carbamidomethyl of Cys. Results were filtered to a maximum false discovery rate of 1%. Quantification was based on the FlashLFQ method embedded in the software.

For the targeted experiment, data were then imported into Skyline software (MacLean et al., 2010; Stergachis et al., 2011) for final processing and evaluation. Quantification was based on the area under the curve of extracted ion chromatograms from the most intense transition per peptide.

### Quantification and statistical analysis

Except where otherwise indicated, all of the presented data are representative results of at least two independent experiments. In all diagrams with error bars, the values correspond to mean values and the bars show either the range of results (in the case of duplicate samples) or standard deviations (SD) (in the case of larger sample numbers).

In fluorescence microscopy, colocalization was determined manually using unsaturated single Z-stack images, by measuring the overlap of signals between channels with constant threshold values. Data are presented as means (%) ± SD in approximately equal numbers of images acquired in at least three independent experiments. The *P* values were calculated using a two-tailed Student’s *t*-test.

Bacterial yields are presented as the means of colony-forming units ± SD. The *P* values were calculated using a two-tailed Student’s *t*-test.

## Supplementary information

Supplementary Figure Legends

Supplementary Figure S1

Supplementary Figure S2

Supplementary Figure S3

Supplementary Figure S4

Supplementary Figure S5

Supplementary Figure S6

Supplementary Figure S7

Supplementary Figure S8

Supplementary Figure S9

Supplementary Figure S10

Supplementary Figure S11

Supplementary Figure S12

Supplementary Figure S13

Supplementary Figure S14

Supplementary Figure S15

Supplementary Figure S16

Supplementary Figure S17

Supplementary Figure S18

Supplementary Figure S19

Reproducibility Checklist form

Author contribution statement

## Data Availability

All data generated or analyzed during this study are included in this published article and its supplementary information files.
