# Supplementary material for: Site-specific ubiquitination of MLKL targets it to endosomes and targets Listeria and Yersinia to the lysosomes
Source: Cell Death Differ. 2022 Jan 9;29(2):306–22. doi: 10.1038/s41418-021-00924-7 (PMC8816944; doi:10.1038/s41418-021-00924-7)
Supplement: Supplementary file 1 — Supplementary Figure Legends [file 41418_2021_924_MOESM1_ESM.pdf]

Supplementary Figure Legends

Supplementary Figure S1. Related to Fig. 1. Representation of data from the experiments shown in Fig. 1, along with assessment of the total amounts of ubiquitinated proteins in each of the samples (which was not included in the main figure owing to space limitations).

Supplementary Figure S2. Related to Fig. 1. Effect of the RIPK3 inhibitor GSK-872 on TBZ-induced ubiquitination of MLKL in HT-29 cells. The assay was performed as in Fig. 1A. GSK-872 was applied at a concentration of 5 μM for 3 h (together with TNF**).**

Supplementary Figure S3. Related to Fig. 1. Certain mutations in MLKL that we we found here to ablate its ubiquitination (T357A/S358A and L162G/L165G) do not do so by affecting its recruitment to the necrosome. TBZ-induced association of MLKL with RIPK3 in MLKL-KD HT-29 cells that express constitutively MLKL or its indicated mutants was determined by western blot analysis following MLKL immunoprecipitation using anti-MLKL antibody.

Supplementary Figure S4. Related to Fig. 2. MS/MS spectra of the MLKL peptides spanning the GG sites (ubiquitination). The analysis was performed with MLKL isolated from HT-29 cells following 3 h of treatment with TBZ. Spectra were identified via a database search using MetaMorpheus (https://pubs.acs.org/doi/abs/10.1021/acs.jproteome.7b00873). Purple: y-fragment ions. Blue: b-fragment ions.

Supplementary Figure S5. Related to Fig. 2. Representation of data from the experiments shown in the corresponding panels in Fig. 2, along with assessment of the total amount of ubiquitinated proteins in each of the samples (which was not included in the main figure owing to space limitations).

Supplementary Figure S6. Related to Fig. 2. Effects of mutational replacement of lysine residues in human MLKL and mouse MLKL by arginine on the effectiveness of necroptotic death induction. A and B. Comparison of the kinetics of cell death induction by TBZ in HT-29 cells knocked out for MLKL, that were reconstituted with inducible wild type

MLKL, or (A) with MLKL in which lysine 230 was replaced by arginine, or (B) with MLKL in which lysine 50 was replaced by arginine.
C. Comparison of the kinetics of cell-death induction by TBZ in MEFs with knocked out MLKL that were reconstituted with inducible wild-type MLKL, or with MLKL in which both lysine residues 50 and 51 were replaced with arginine.
Western blot analyses confirming the expression of equal amounts of human and mouse MLKL in the compared cells are shown in the insets.
(Each of the presented experiments was performed twice, each time with duplicate samples.)

Supplementary Figure S7. Related to Fig. 2. Effect of mutations of lysines 50 and 51 in mouse MLKL on its ubiquitination and on death induction by it in L929 cells in response to double-stranded RNA. Wild-type MLKL and its K50, 50R mutant were expressed inducibly in MLKL-KO L929 cells. The cells were treated for 16h with β interferon (50 U/ml), and then for the indicated times with poly I:C (25 μg/ml) and zVAD (25 μM). The extents of cell death and of MLKL ubiquitination were assessed as described in Materials and Methods.

Supplementary Figure S8. Related to Fig. 3. Triggering the phosphorylation of MLKL in HT-29 cells induces its association, but not that of its K50R mutant, with endosomal membranes. Assessment of the association of MLKL and of its phosphorylated form with endosomal membranes in MLKL KO HT-29 cells expressing either wild-type MLKL or its K50R mutant, compared with their amounts in the whole cell lysate and in the cytosol. Endosomal membranes were isolated by a discontinuous sucrose gradient method (60). Samples (15 μg) of the proteins were subjected to western blot analysis. This experiment was carried out twice, with identical results.

Supplementary Figure S9. Related to Fig. 3. TBZ-treatment induces association of MLKL and ubiquitin with endosomes, but not with the endoplasmic reticulum. Comparative immunocytological analysis of the extent of association of MLKL and ubiquitin with (A) early endosomes and (B) the endoplasmic reticulum in HT-29 cells following treatment for 3 h with TBZ. Green – MLKL; red – early endosomes (in A) or endoplasmic reticulum (in B); blue – ubiquitin; cyan – MLKL + ubiquitin; magenta – early endosomes (in A) or endoplasmic reticulum (in B) + ubiquitin; yellow – MLKL + early endosomes (in A) or

MLKL + endoplasmic reticulum (in B); white (and arrow) – MLKL + early endosomes + ubiquitin. Early endosomes and the endoplasmic reticulum were visualized using CellLight Early Endosomes-GFP (10586) and CellLight ER-GFP (C10590), and MLKL and ubiquitin were visualized by applications of their specific antibodies. Fidelity of the anti-MLKL antibody used for immunostaining (ab184718 from Abcam) was reconfirmed by comparing the immunostaining of wild-type and MLKL KO HT-29 cells.

Supplementary Figure S10. Related to Fig. 3. Confirmation of MLKL colocalization with ubiquitin in endosomes by bimolecular fluorescence complementation (BiFC) analysis. BiFC analysis of the location of ubiquitinated MLKL in control (A) and TBZ-treated (B) HT-29 cells by expression of MLKL and of ubiquitin fused to C-terminal and N-terminal fragments of YFP, respectively, was conducted as described in Materials and Methods. TBZ treatment was for 3 h. Cyan – MLKL + ubiquitin; magenta – Rab7; white (and arrow) – MLKL + ubiquitin + Rab7. In this experiment, Rab7 was detected by immunostaining.

Supplementary Figure S11. Related to Fig. 4. Representation of data from the experiments shown in the corresponding panels in Fig. 4, along with assessment of the total amounts of ubiquitinated proteins in each of the samples (which was not included in the main figure owing to space limitations).

Supplementary Figure S12. Related to Fig. 4. In response to TBZ, ITCH binds inducibly to MLKL in HT-29 cells, and this binding is not affected by replacement of lysine 50 with arginine. (A). Assessment of associations of the endogenous proteins. (B) Assessment of association of the endogenous ITCH with the wild type, or with the K50R mutant MLKL, fused to the Strep-tactin tag and expressed inducibly in MLKL KO cells.

Supplementary Figure S13. Related to Fig. 4. Defining the region within MLKL that binds ITCH. ITCH, fused C-terminally to the Flag tag, and the indicated deletion mutants of MLKL fused C-terminally to the double strep tag (STR), were expressed by transient transfection in HEK 293T cells. Coprecipitation of the proteins was assessed by WB.

Supplementary Figure S14. Related to Fig. 4. Recombinant human ITCH binds to both recombinant human and mouse MLKL and ubiquitinates them. (A) Assessment of

binding of ITCH to MLKL. Human and mouse MLKL, fused N-terminally to the 6xHis-SUMO tag (60 ng/ml), produced *in E. coli,* were incubated for 2 h at 4^0C with recombinant human ITCH (600 ng/ml), in 1% NP-40 lysis buffer containing protein-G Sepharose (30 μl/ml). Following centrifugation for 5 min at 1000 × g, NI-NTA His-BIND resin was added to the supernatant (50 μl/ml), and after further incubation for 30 min at 4^0C the resin was spun down for 1 min at 1000 × g and washed five times with the lysis buffer. The proteins bound to it were analyzed by SDS−PAGE and western blotting. (B) *In-vitro* ubiquitination of the recombinant MLKL proteins by the ITCH was assessed as described in Materials and Methods.

Supplementary Figure S15. Related to Fig. 4. Knockout of ITCH facilitates the induction of necroptosis. Comparison of the kinetics of cell-death induction by TBZ in wild-type and ITCH KO (A) HT-29 cells and (B) in MEFs. This experiment was done twice.

Supplementary Figure S16. Related to Fig. 5. Comparison of the amounts of wild-type and mutant MLKL in the cells used in the experiments presented in Fig. 5A and B.

Supplementary Figure S17. Related to Fig. 5. Infection of HT-29 cells with *Listeria* does not impose MLKL oligomerization or ubiquitination . Assessment of MLKL ubiquitination (top) and oligomerization (middle) in HT-29 cells in response to their infection with *Listeria*, and in response to their infection with *Listeria* combined with TBZ treatment for the last 2 h of the infection period.

Supplementary Figure S18. Related to Fig. 7. Knockout of ATG5 in HT-29 cells does not affect the extent of MLKL ubiquitination in response to TBZ treatment or the inhibitory effects of MLKL on the growth of *Listeria* and *Yersinia.* A. Assessment of the effect of KO of ATG5 on MLKL ubiquitination in HT-29 cells. The arrowhead points to phosphorylated MLKL.

B, C. Assessments of the effects of KO of ATG5 on the amounts of viable *Listeria* (B) and *Yersinia* (C) in HT-29 cells, and on their modulation by TBZ treatment. Three independent tests were performed. (n=6)

Supplementary Figure S19. Related to Figs 5, 6 and 7. Diagrammatic presentations of the protocols of bacterial infection of cells.
