# Supplementary figures and images for: Site-specific ubiquitination of MLKL targets it to endosomes and targets Listeria and Yersinia to the lysosomes
Source: Cell Death Differ. 2022 Jan 9;29(2):306–22. doi: 10.1038/s41418-021-00924-7 (PMC8816944; doi:10.1038/s41418-021-00924-7)

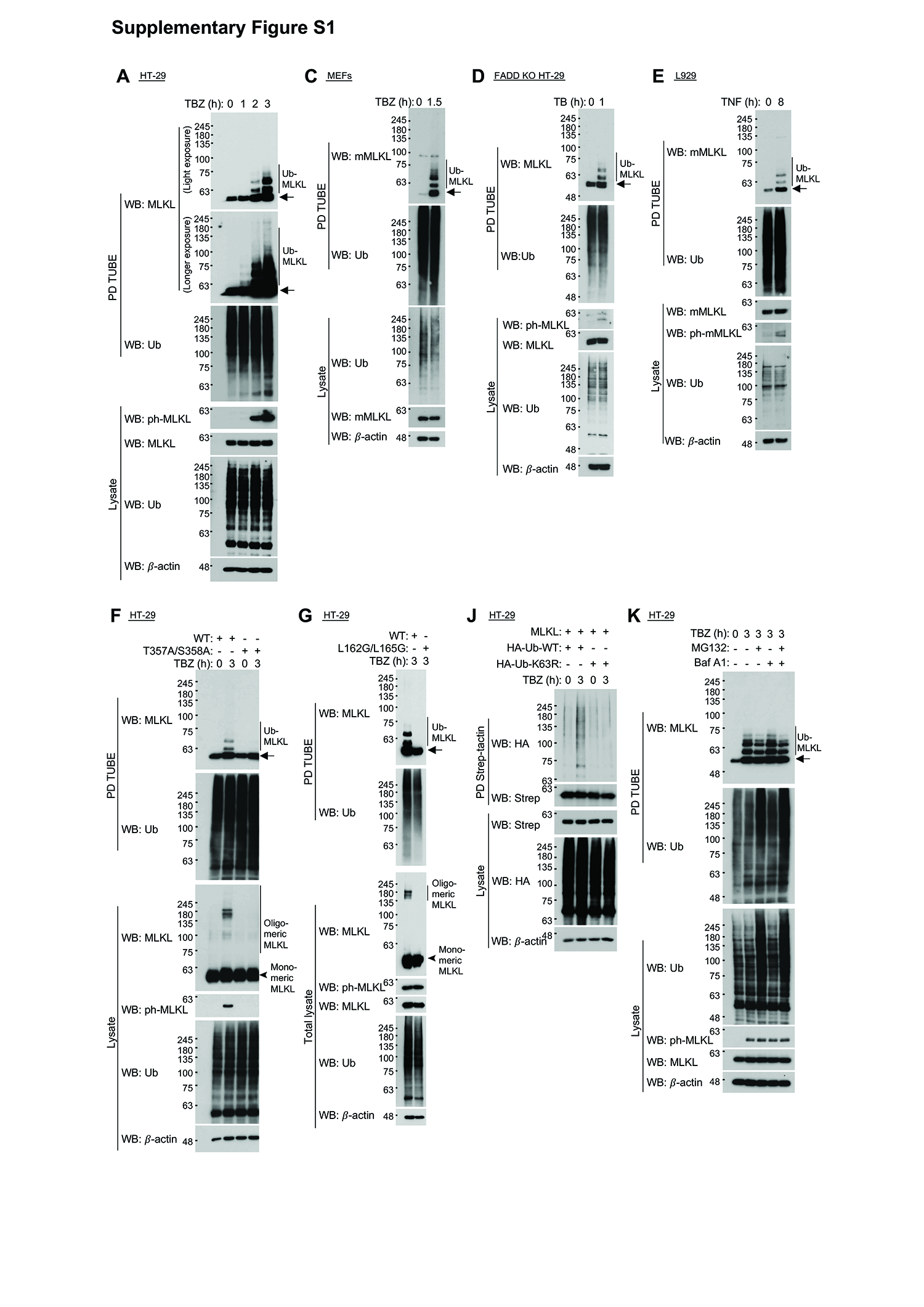

Supplement: Supplementary file 2 — Supplementary Figure S1 [file 41418_2021_924_MOESM2_ESM.tif]

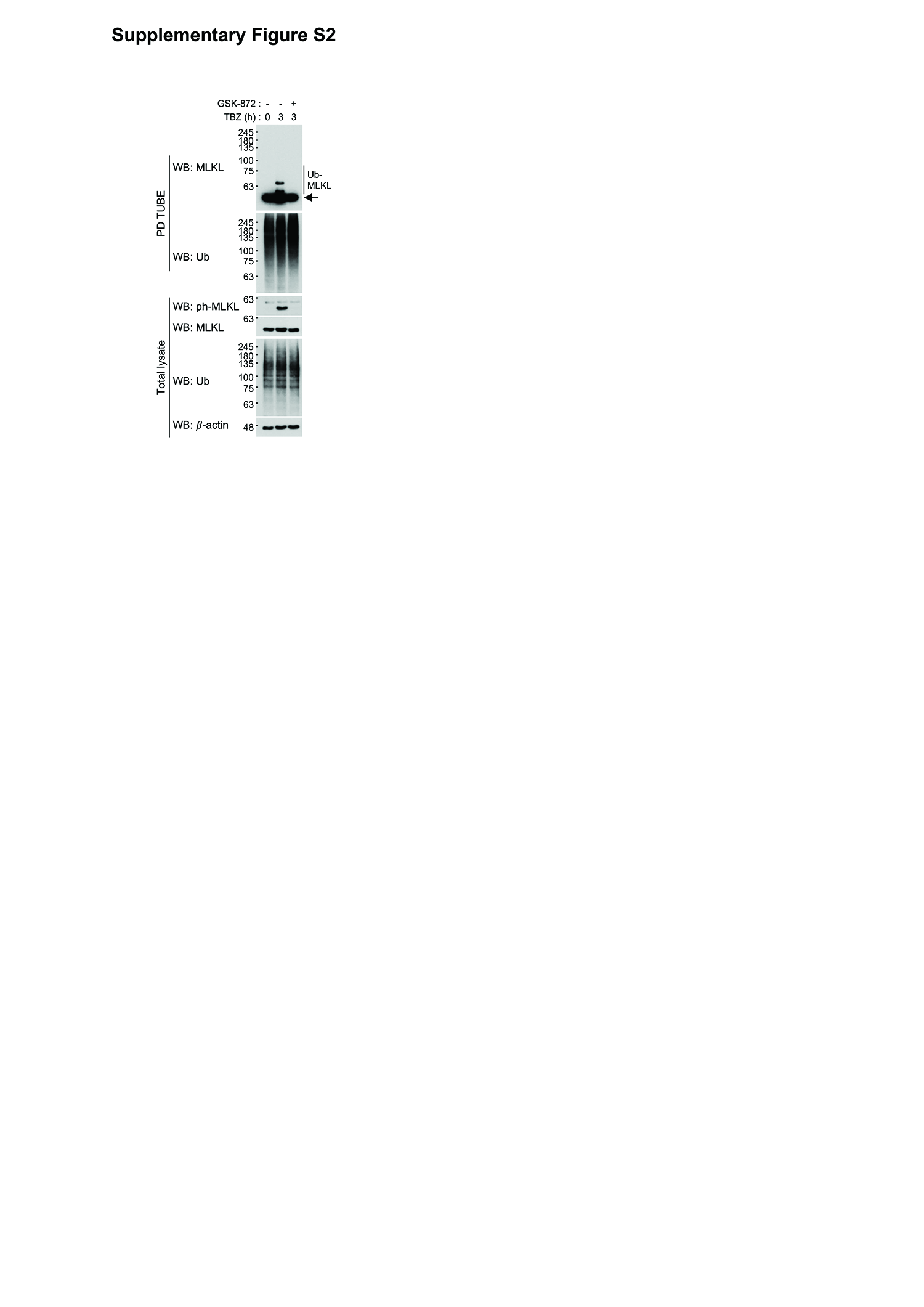

Supplement: Supplementary file 3 — Supplementary Figure S2 [file 41418_2021_924_MOESM3_ESM.tif]

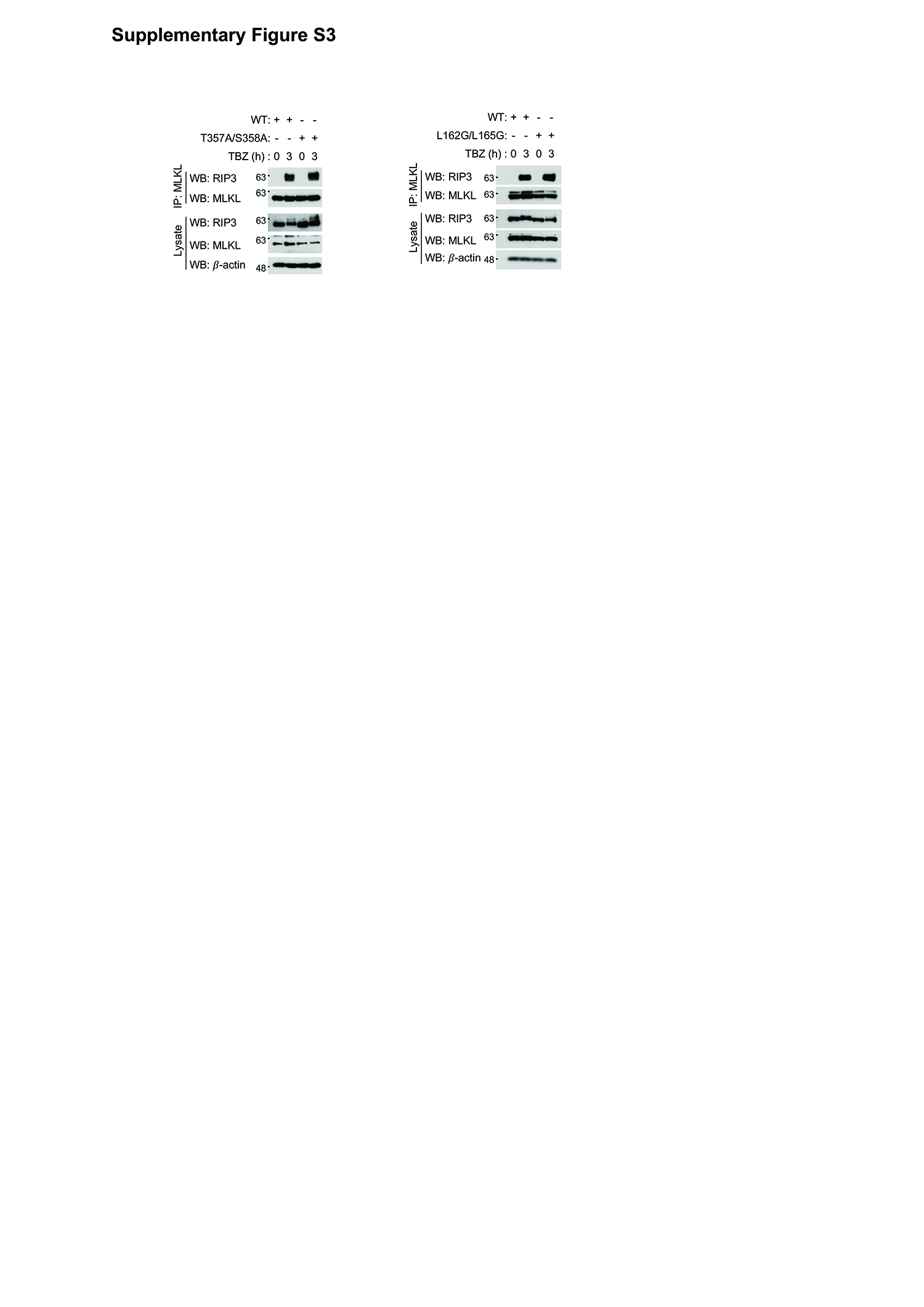

Supplement: Supplementary file 4 — Supplementary Figure S3 [file 41418_2021_924_MOESM4_ESM.tif]

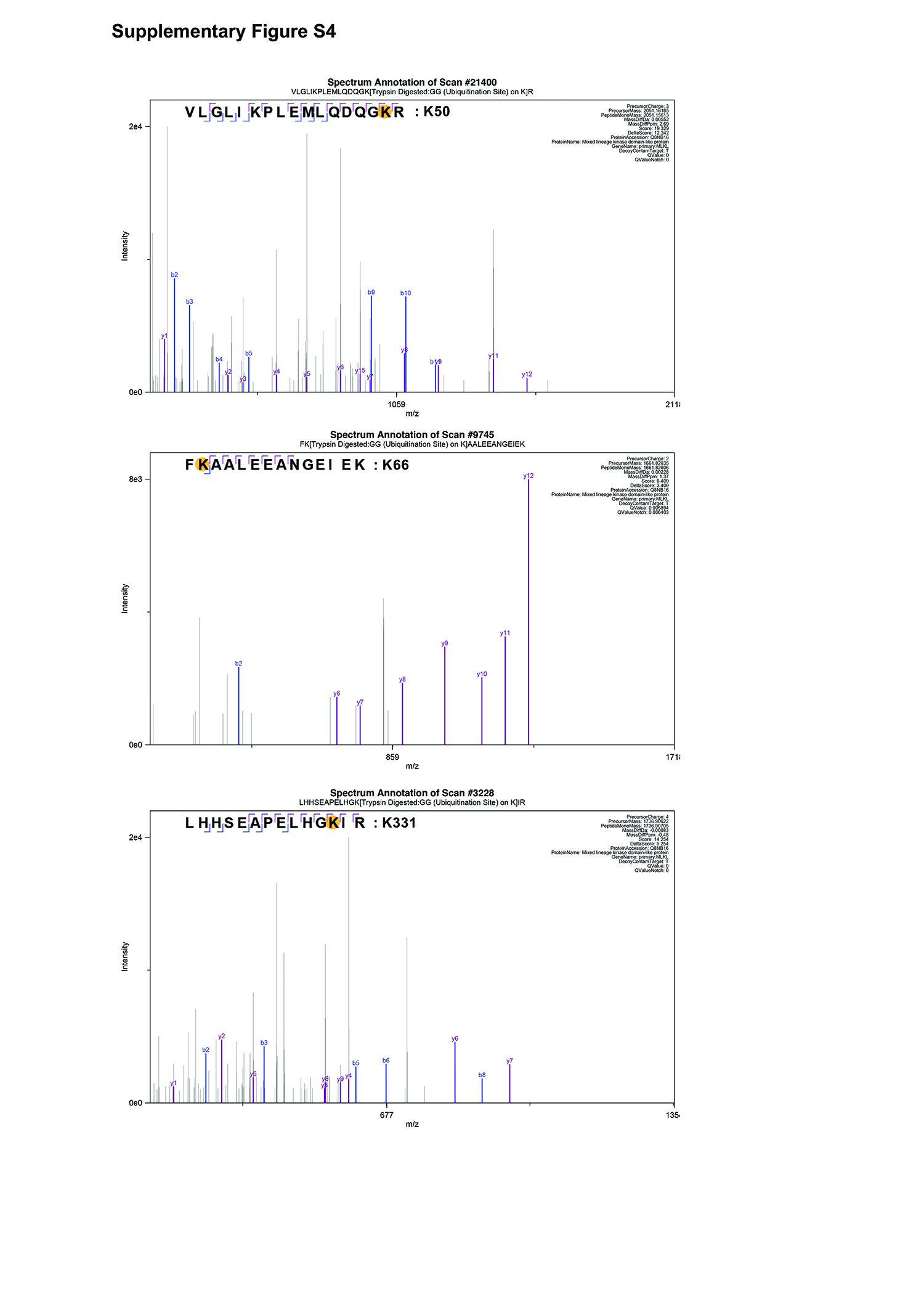

Supplement: Supplementary file 5 — Supplementary Figure S4 [file 41418_2021_924_MOESM5_ESM.tif]

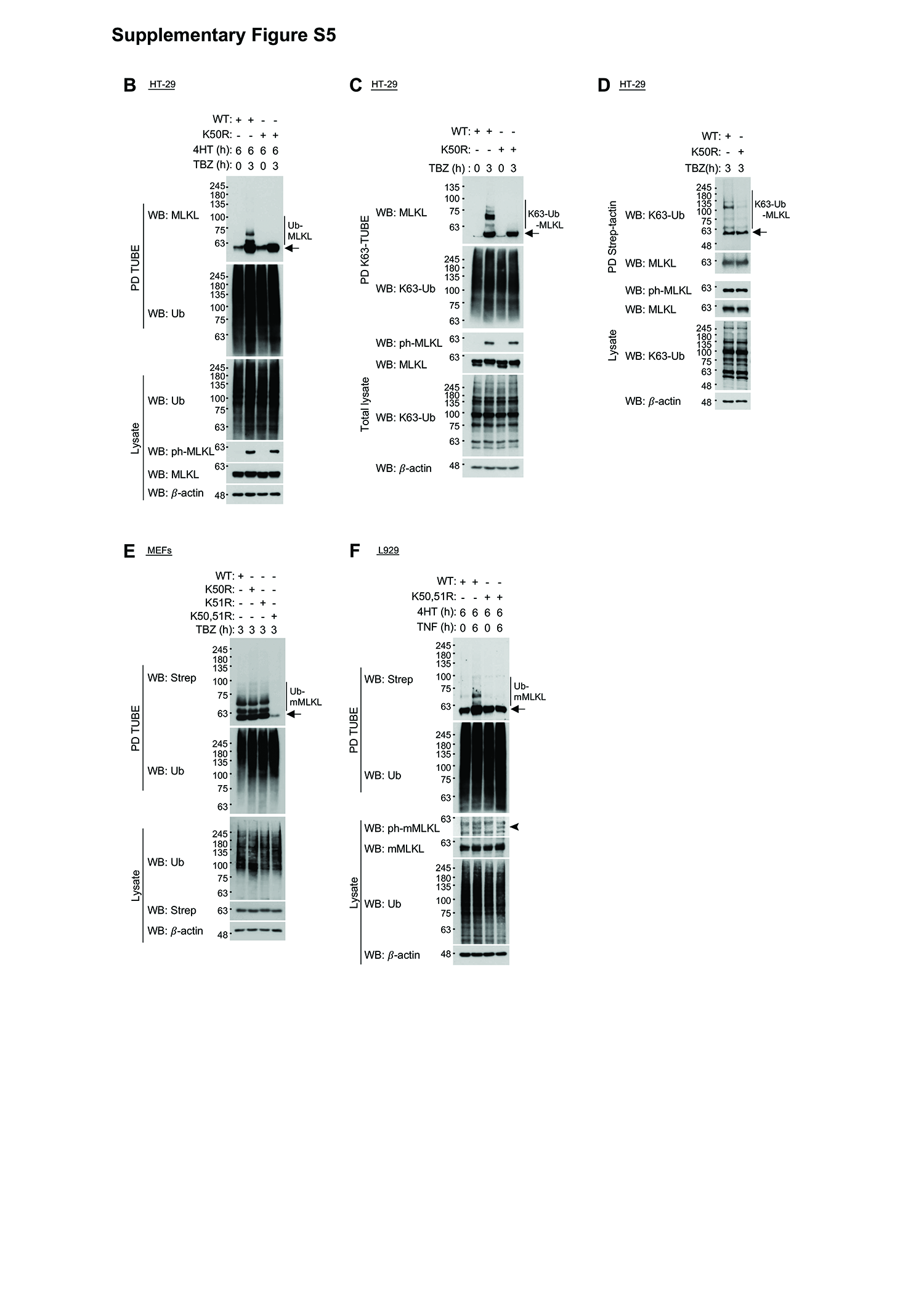

Supplement: Supplementary file 6 — Supplementary Figure S5 [file 41418_2021_924_MOESM6_ESM.tif]

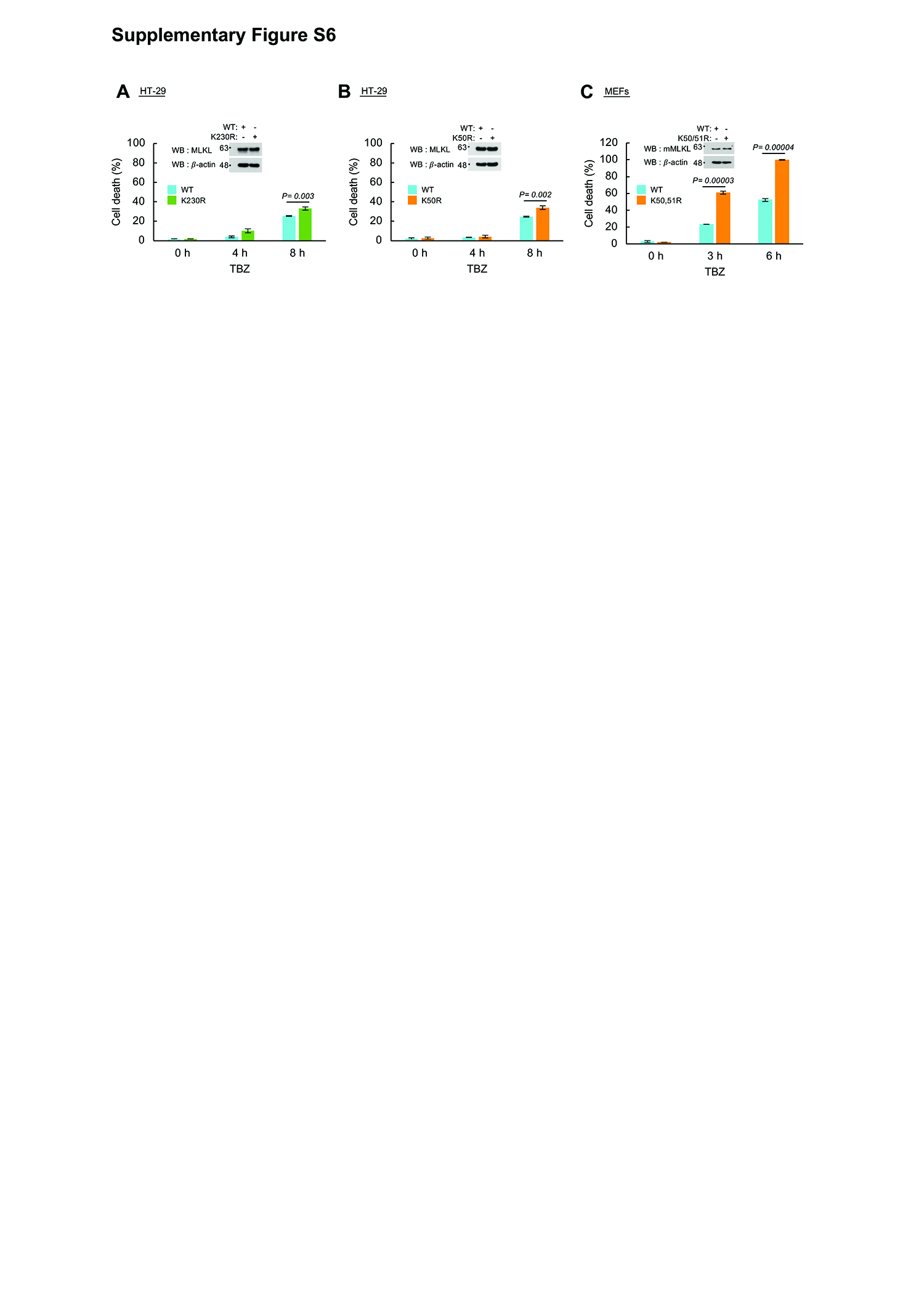

Supplement: Supplementary file 7 — Supplementary Figure S6 [file 41418_2021_924_MOESM7_ESM.tif]

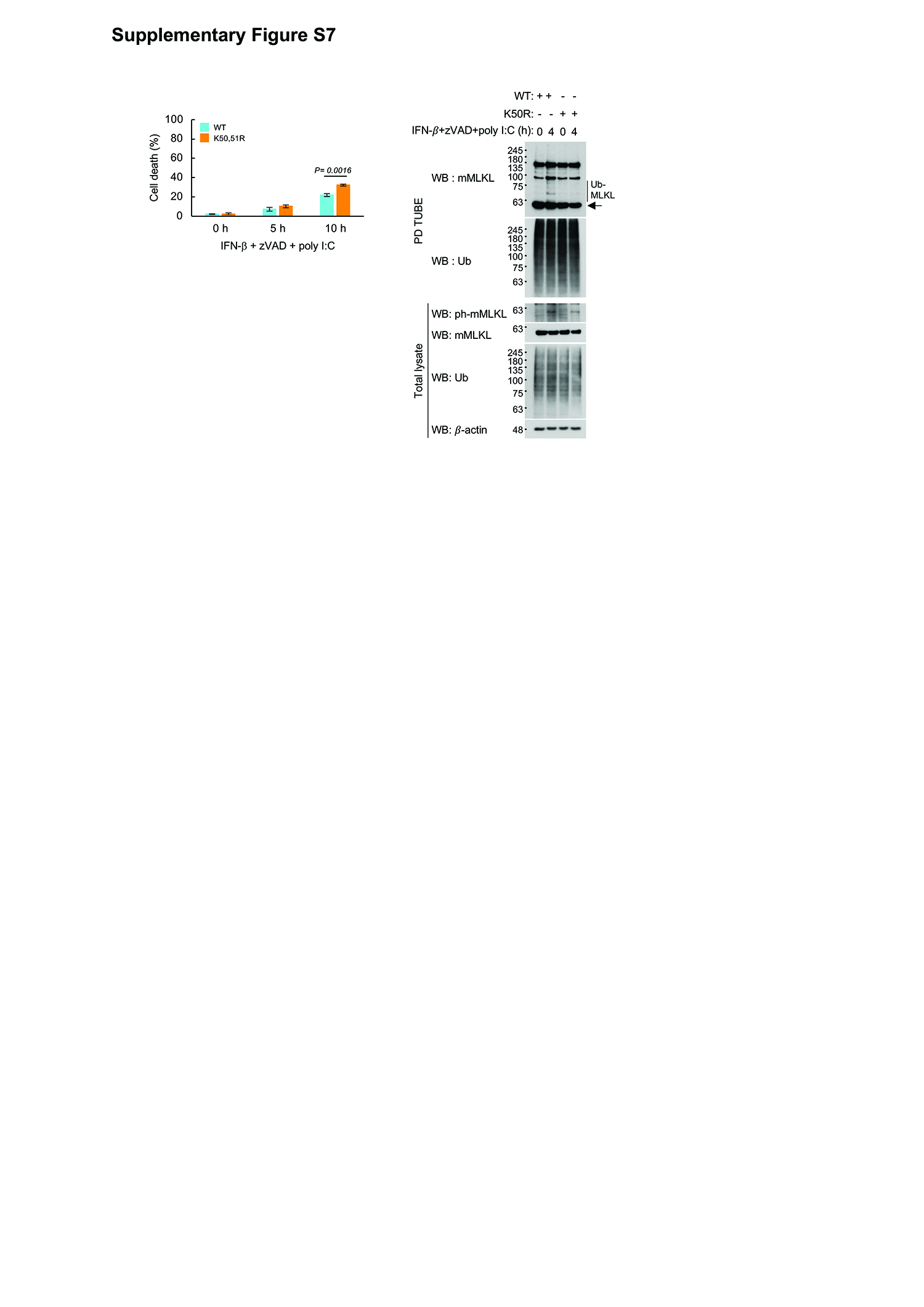

Supplement: Supplementary file 8 — Supplementary Figure S7 [file 41418_2021_924_MOESM8_ESM.tif]

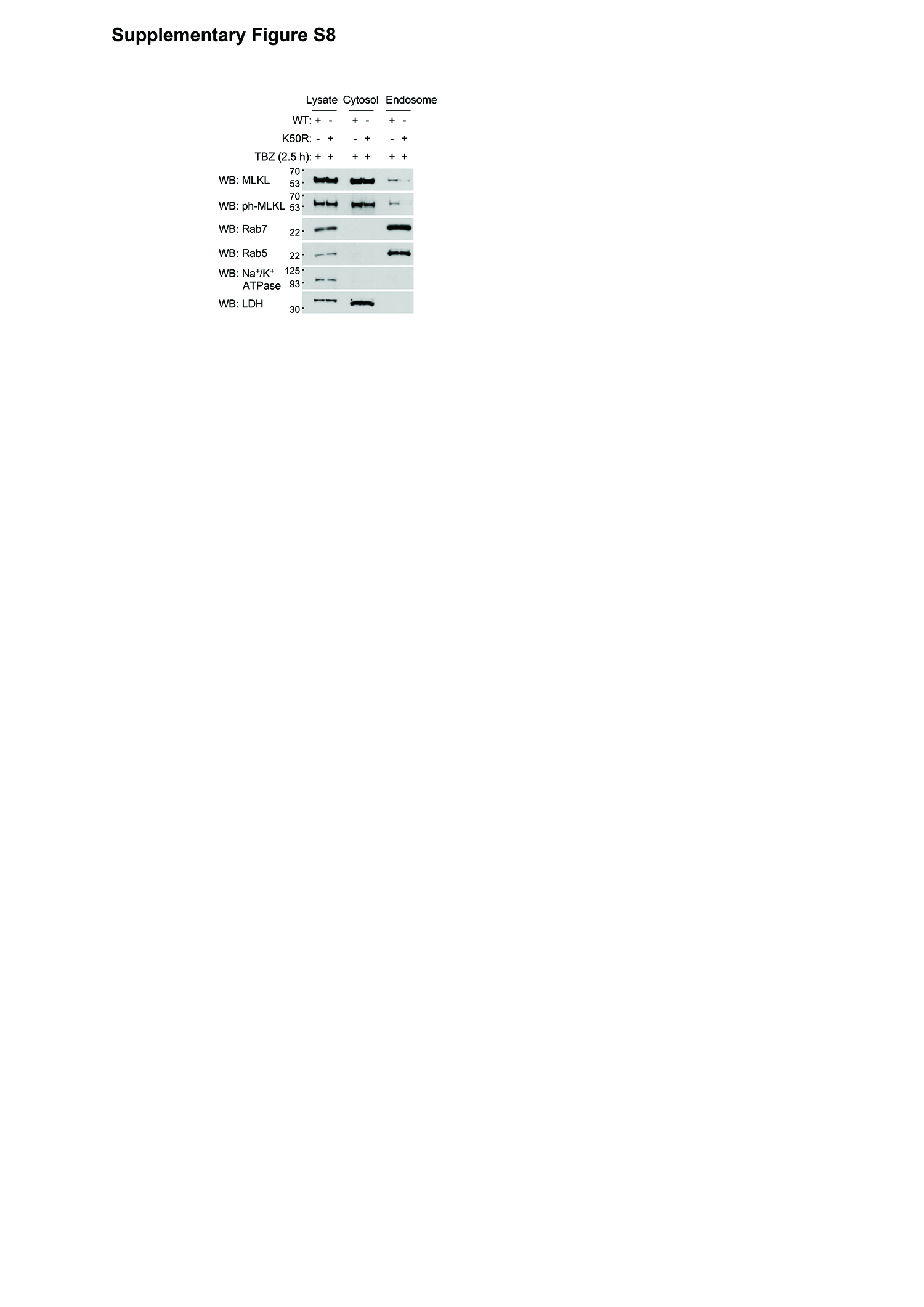

Supplement: Supplementary file 9 — Supplementary Figure S8 [file 41418_2021_924_MOESM9_ESM.tif]

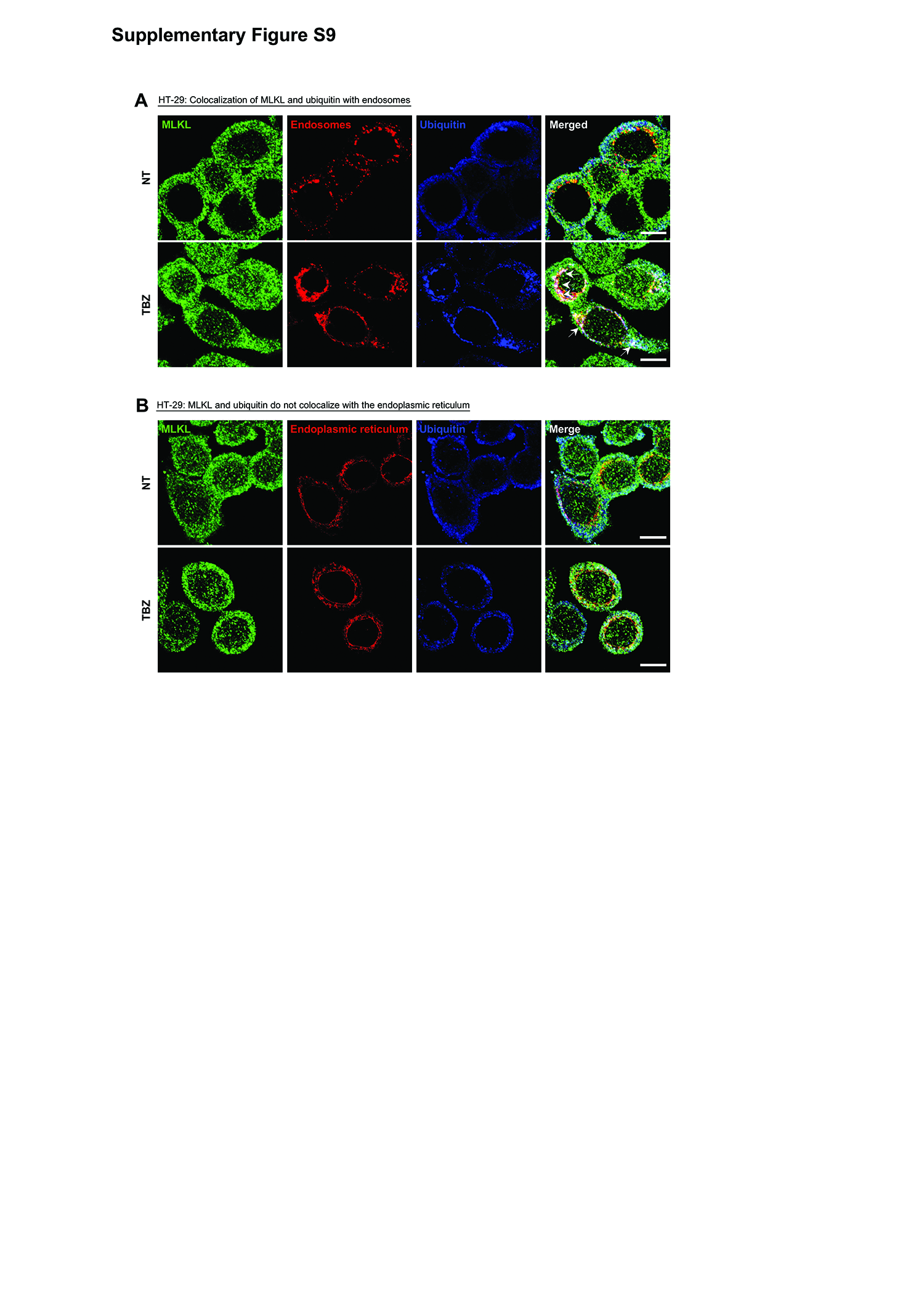

Supplement: Supplementary file 10 — Supplementary Figure S9 [file 41418_2021_924_MOESM10_ESM.tif]

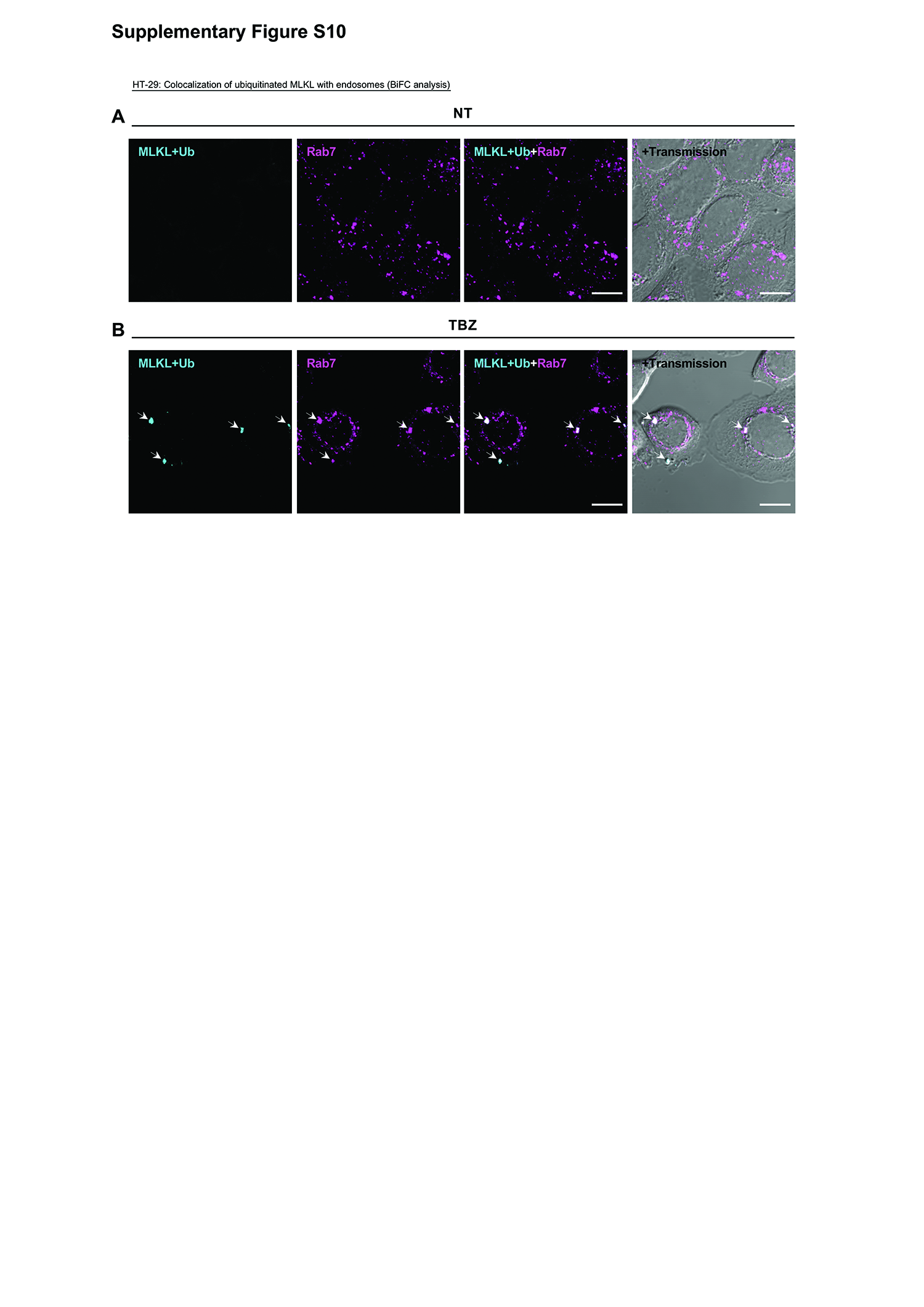

Supplement: Supplementary file 11 — Supplementary Figure S10 [file 41418_2021_924_MOESM11_ESM.tif]

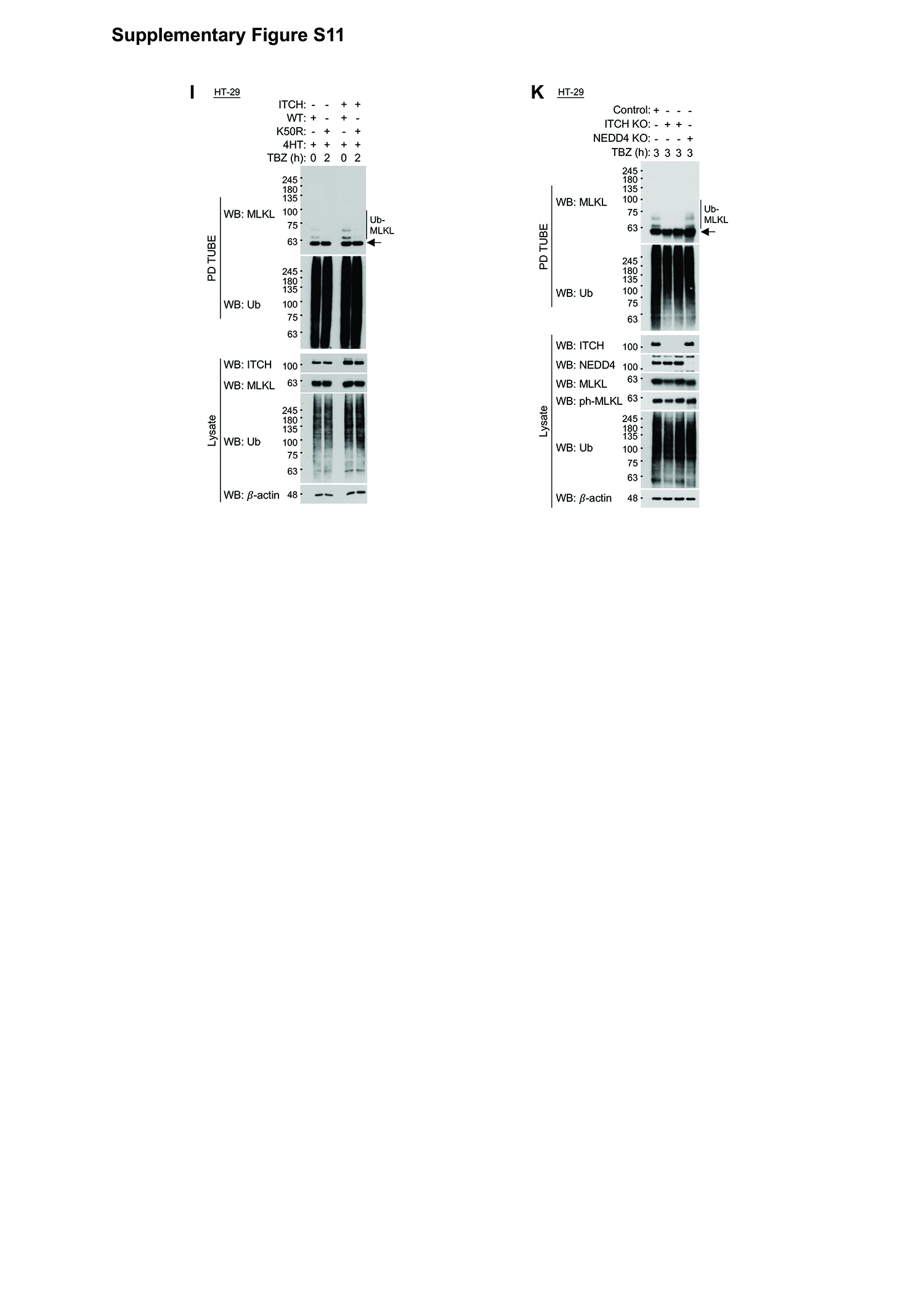

Supplement: Supplementary file 12 — Supplementary Figure S11 [file 41418_2021_924_MOESM12_ESM.tif]

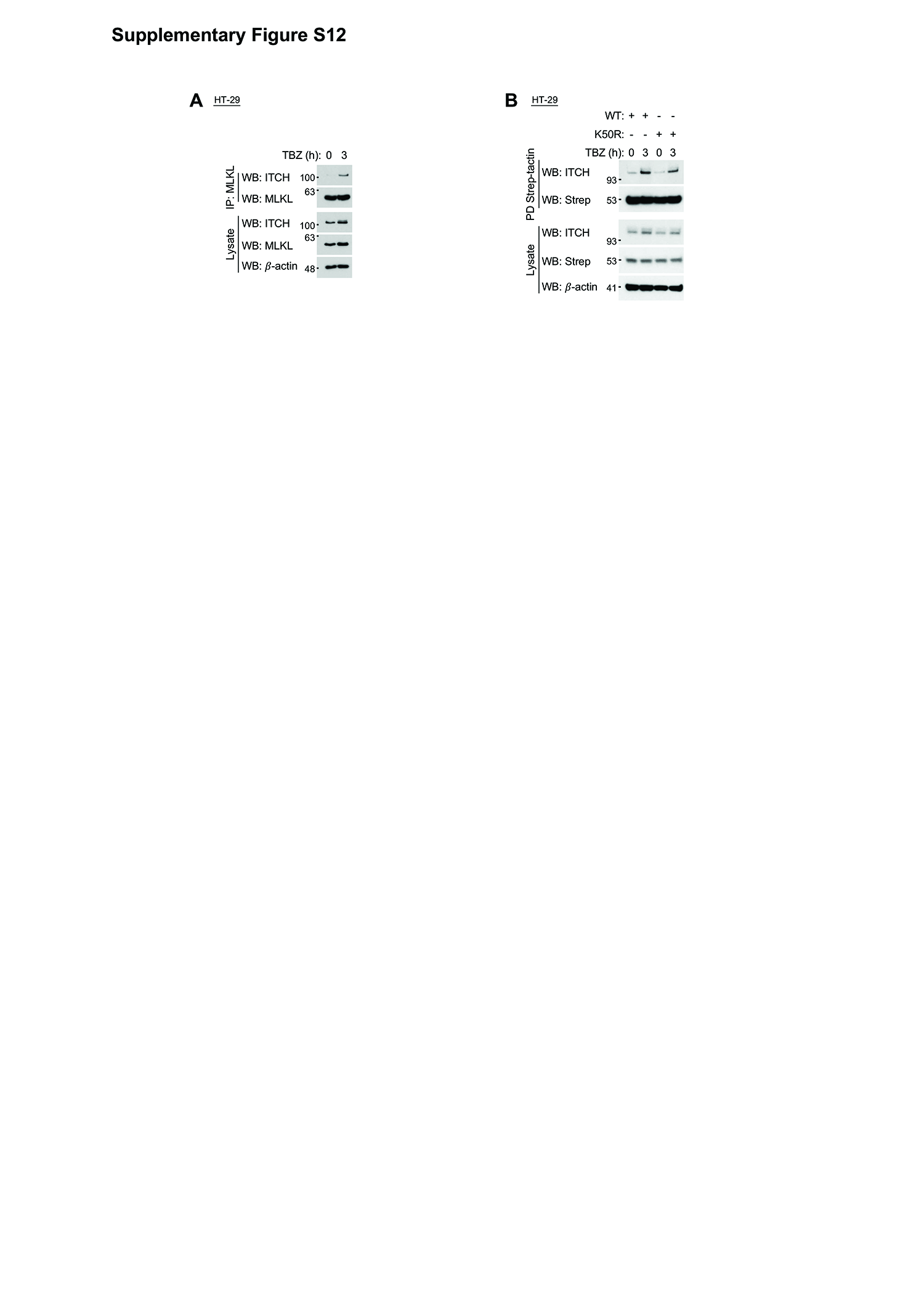

Supplement: Supplementary file 13 — Supplementary Figure S12 [file 41418_2021_924_MOESM13_ESM.tif]

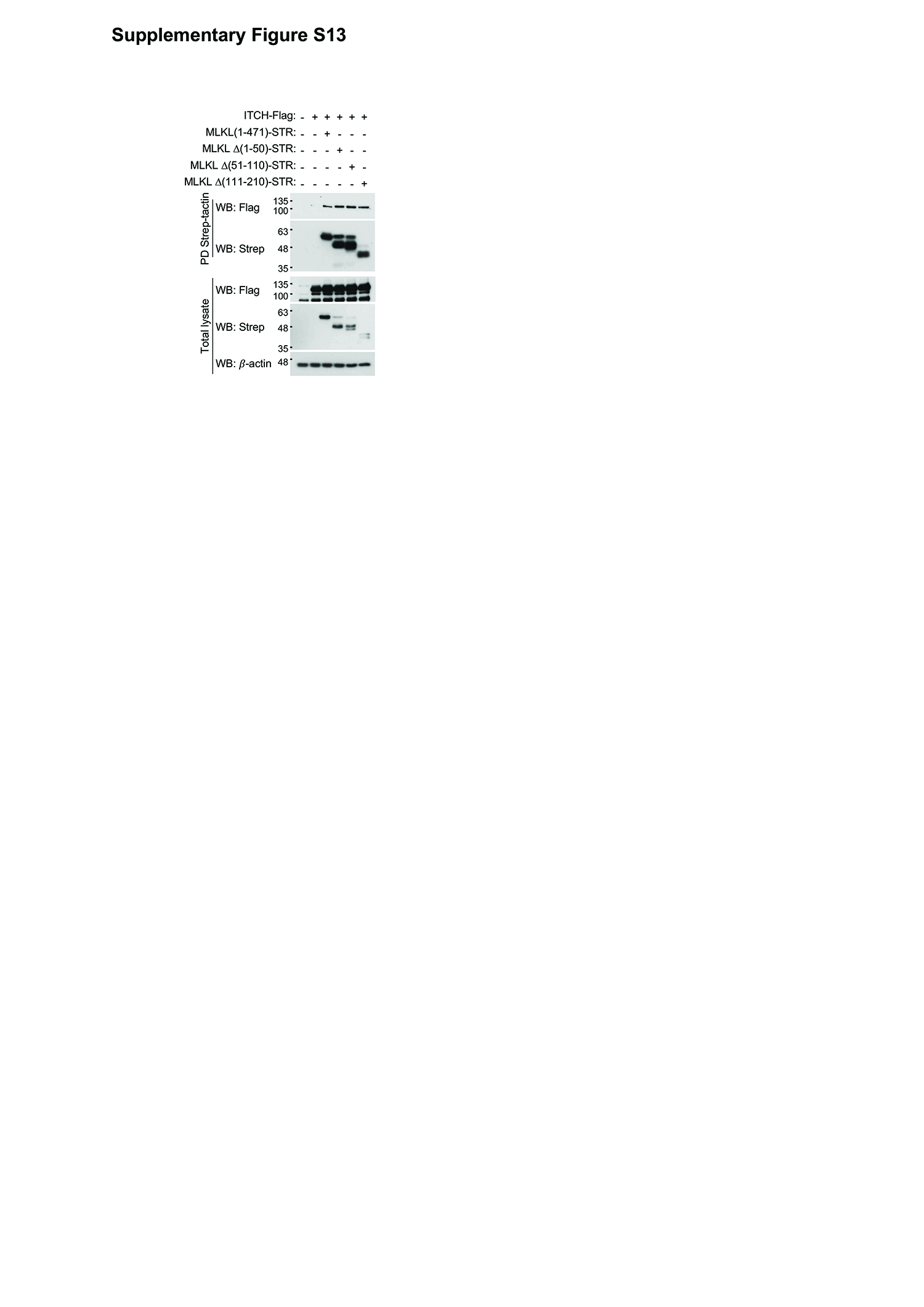

Supplement: Supplementary file 14 — Supplementary Figure S13 [file 41418_2021_924_MOESM14_ESM.tif]

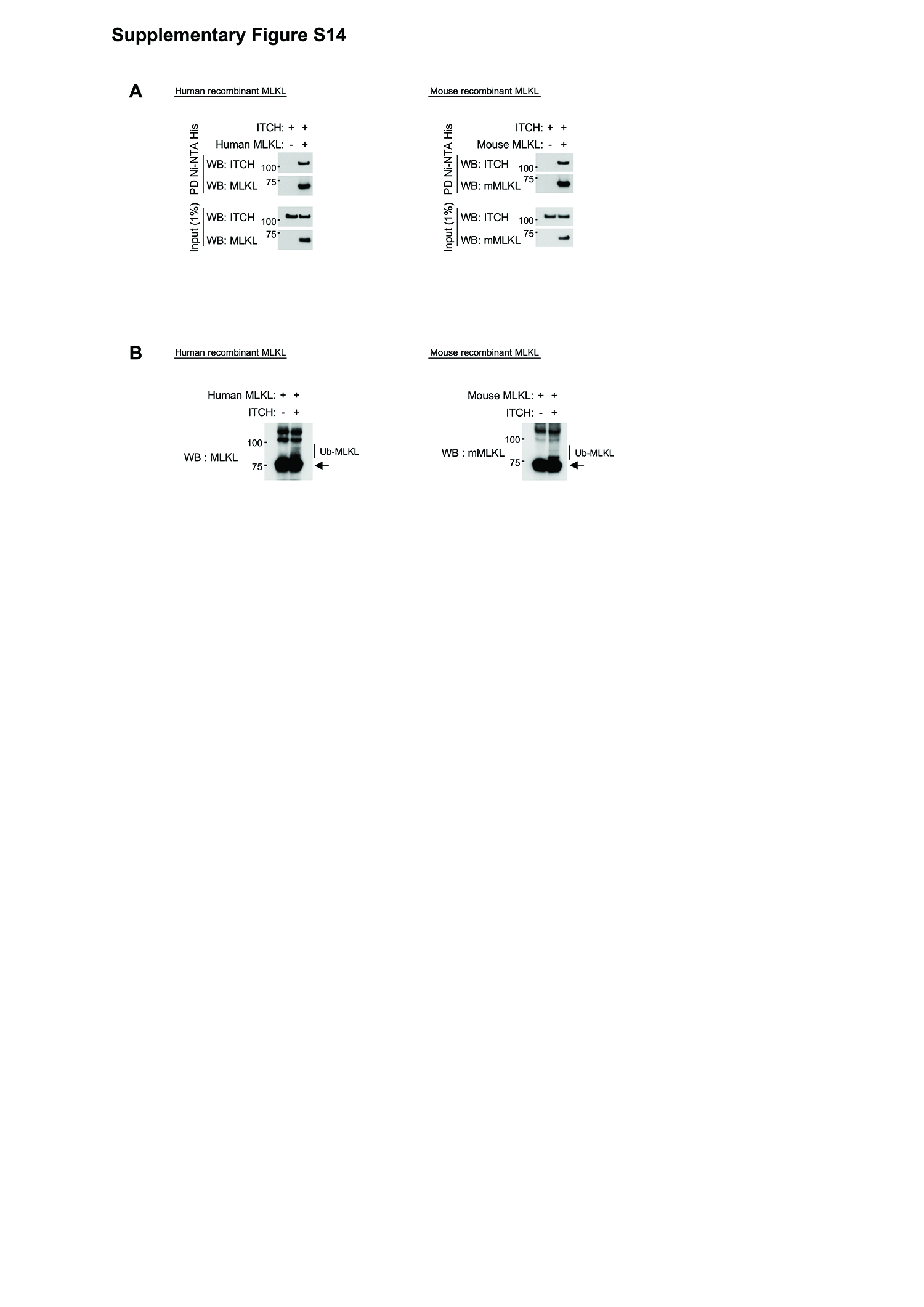

Supplement: Supplementary file 15 — Supplementary Figure S14 [file 41418_2021_924_MOESM15_ESM.tif]

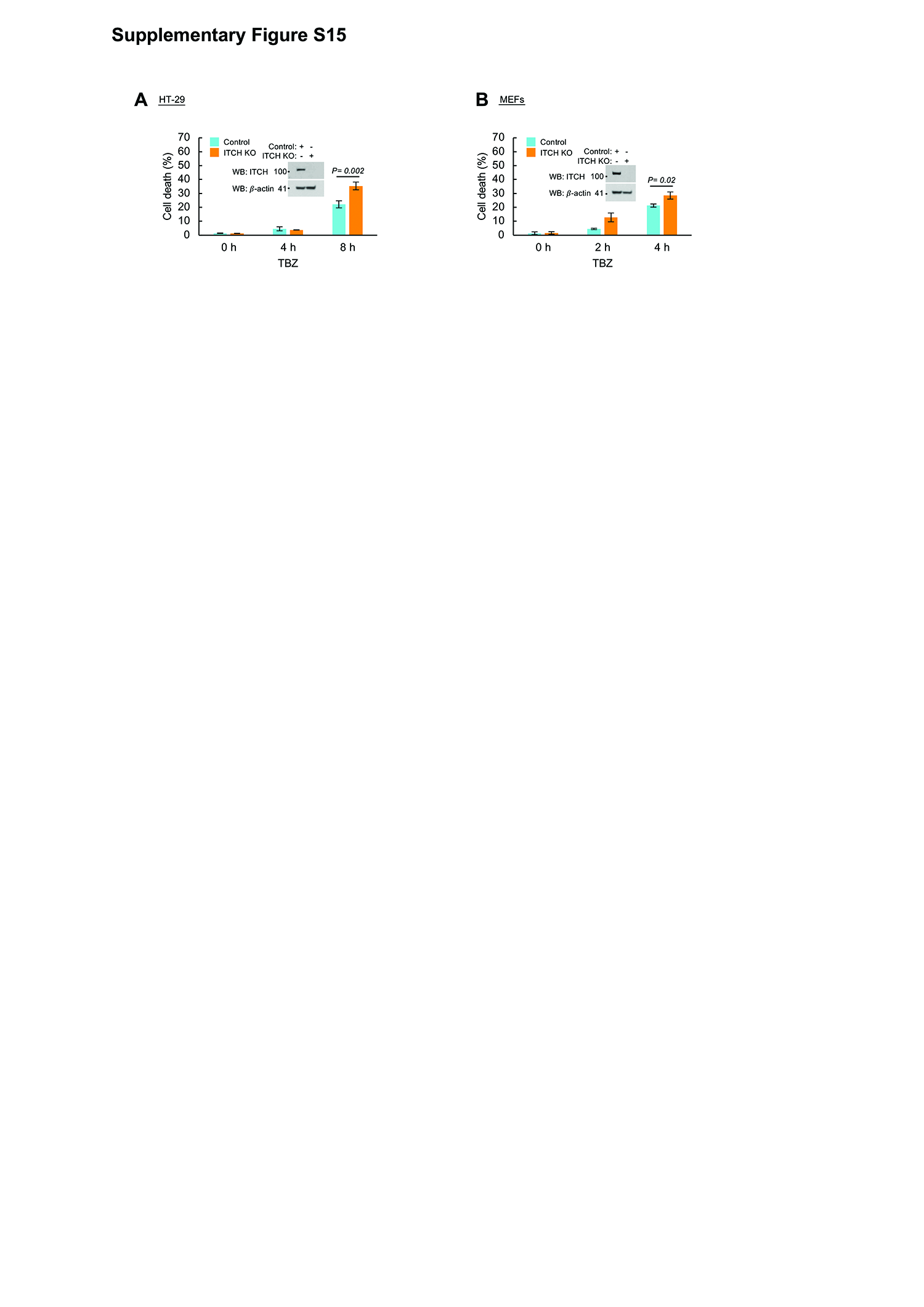

Supplement: Supplementary file 16 — Supplementary Figure S15 [file 41418_2021_924_MOESM16_ESM.tif]

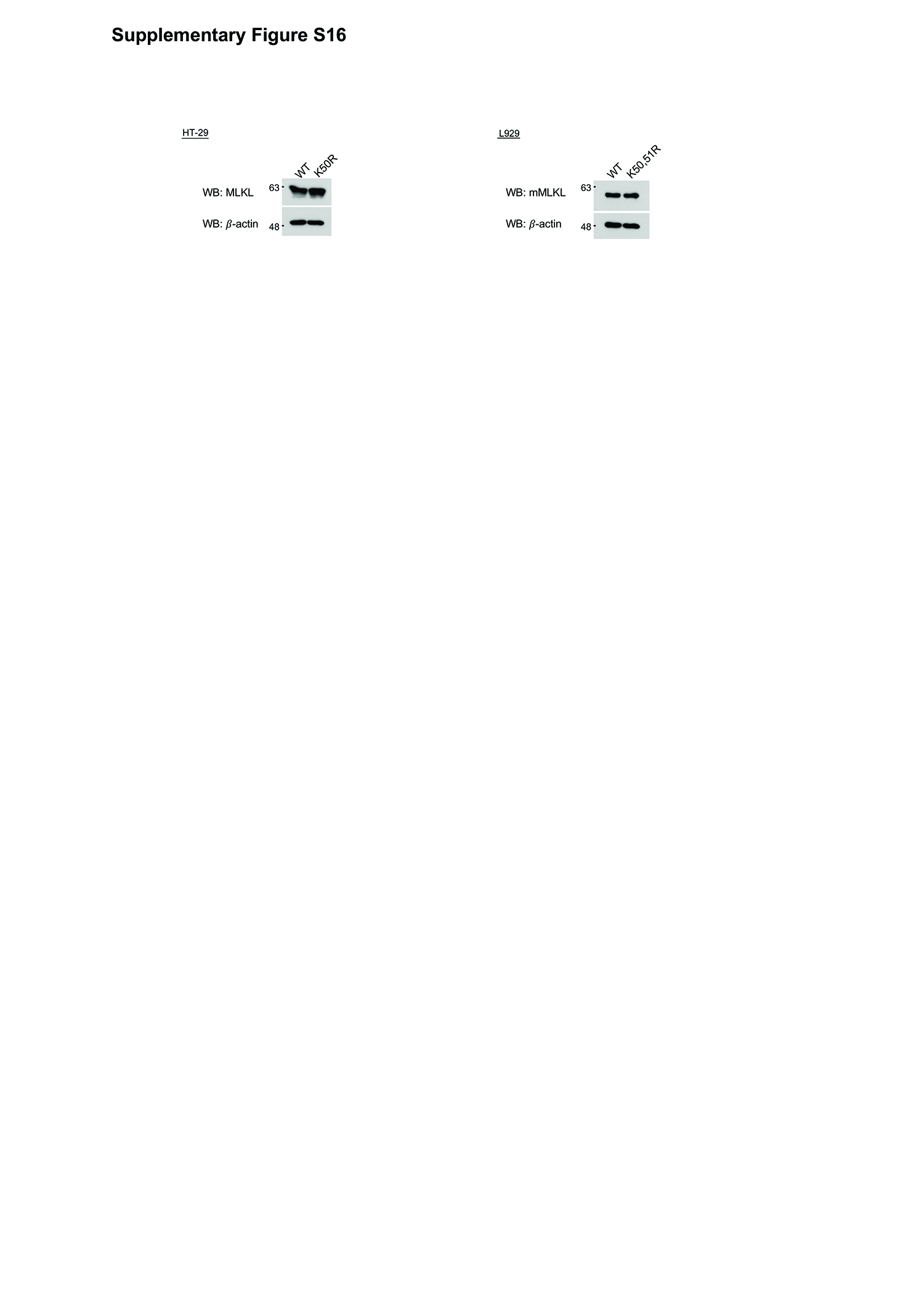

Supplement: Supplementary file 17 — Supplementary Figure S16 [file 41418_2021_924_MOESM17_ESM.tif]

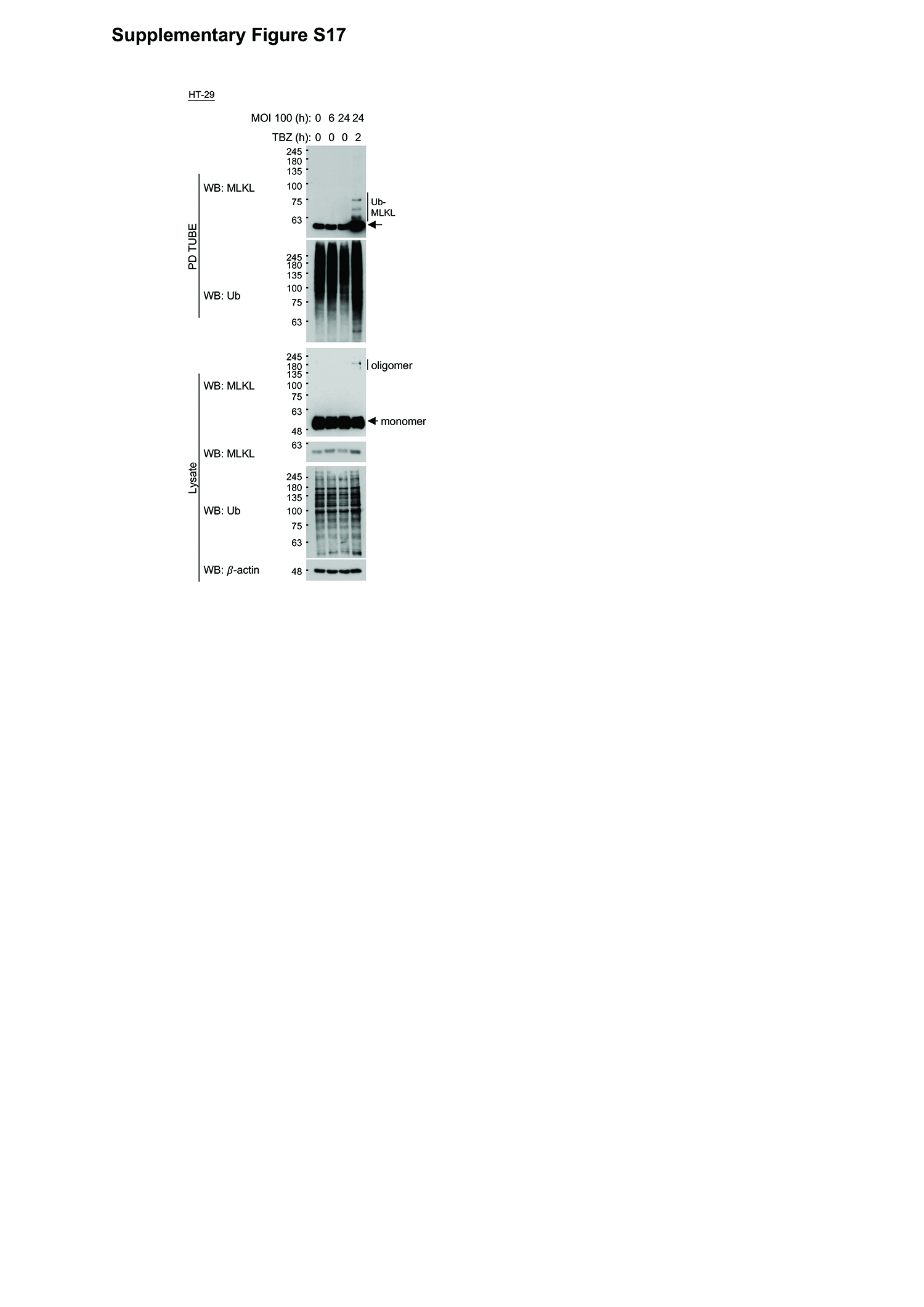

Supplement: Supplementary file 18 — Supplementary Figure S17 [file 41418_2021_924_MOESM18_ESM.tif]

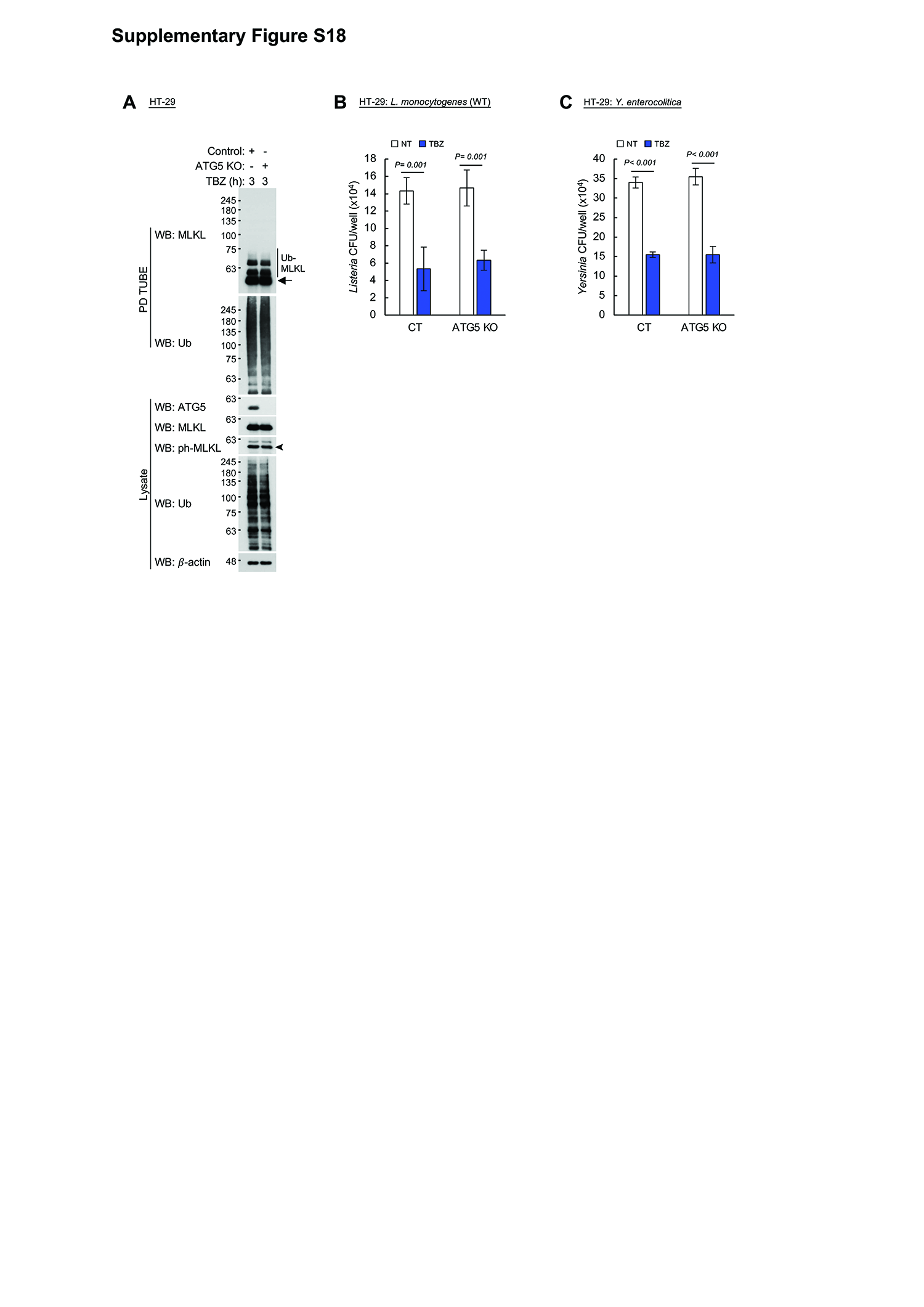

Supplement: Supplementary file 19 — Supplementary Figure S18 [file 41418_2021_924_MOESM19_ESM.tif]

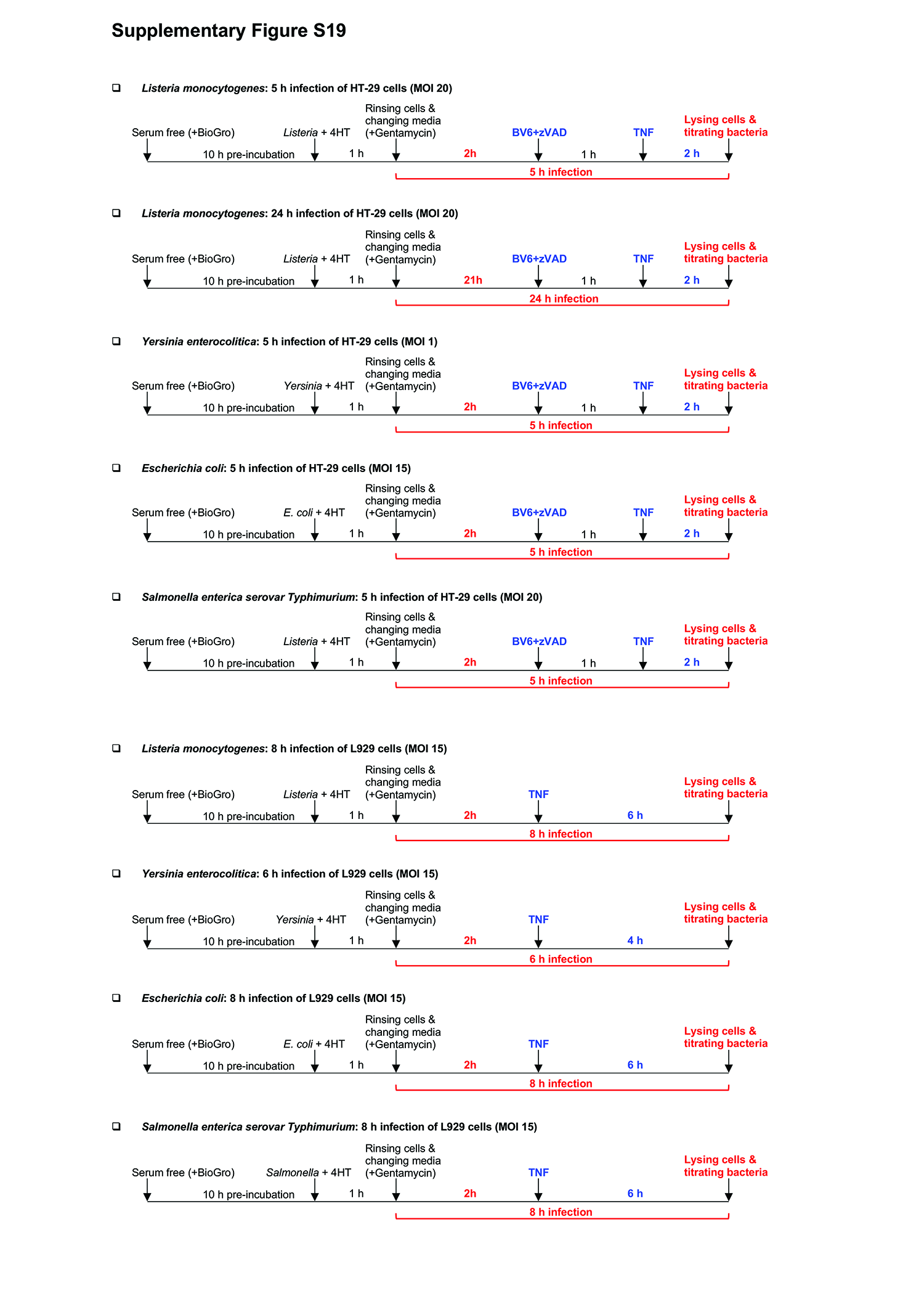

Supplement: Supplementary file 20 — Supplementary Figure S19 [file 41418_2021_924_MOESM20_ESM.tif]
