# Supplementary material for: Site-specific ubiquitination of MLKL targets it to endosomes and targets Listeria and Yersinia to the lysosomes
Source: Cell Death Differ. 2022 Jan 9;29(2):306–22. doi: 10.1038/s41418-021-00924-7 (PMC8816944; doi:10.1038/s41418-021-00924-7)
Supplement: Supplementary file 22 — Author contribution statement [file 41418_2021_924_MOESM22_ESM.pdf]

DECLARATION OF CONTRIBUTIONS TO ARTICLE

ADMC

Manuscript Number: CDD-21-1659R

Journal Name: *Cell Death & Differentiation* (the 'Journal')

Proposed Title of the Contribution: Site-specific ubiquitination of MLKL targets it to endosomes and targets Listeria and Yersinia to the lysosomes (the 'Contribution)

Author(s): Seongmin Yoon, Konstantin Bogdanov, David Wallach (the 'Authors')

For all *CDD* articles, each person named as an author in the published version must be able to show he or she has contributed substantially to the article.

Authorship credit should be based on 1) substantial contributions to conception and design, acquisition of data, or analysis and interpretation of data; 2) drafting the article or revising it critically for important intellectual content; and 3) final approval of the version to be published. Authors should meet conditions 1, 2 and 3.

Any person who cannot be shown to have made a substantial contribution to the article cannot be listed as an author in the final version. The name of any person who is deemed to have made a minor contribution can, however, appear in the Acknowledgments section of the article.

Please complete the table below to indicate the contributions of all named authors to the manuscript.

Author Full Name:	Specification of Contribution to the Manuscript:
Seongmin Yoon	Responsible for project conception, data analysis and manuscript editing.
Konstantin Bogdanov	DNA construct generation
David Wallach	Responsible for project conception, data analysis and manuscript editing. Supervised the study and wrote the manuscript

Please complete the table below to indicate the contributions of all named authors to the figures.

Figure 1:

D. W. – conception, supervision, data analysis
S. Y. – conception, performance of the experiments, data analysis
K.B. – DNA construct generation and quality control

Figure 2:

D. W. – conception, supervision, data analysis, figure design
S. Y. – conception, performance of the experiments, data analysis, figure design
K.B. – DNA construct generation and quality control

Figure 3:

D. W. – conception, supervision, data analysis, figure design
S. Y. – conception, performance of the experiments, data analysis
K.B. – DNA construct generation and quality control, figure design

Figure 4:

D. W. – conception, supervision, data analysis, figure design
S. Y. – conception, performance of the experiments, data analysis
K.B. – DNA construct generation and quality control, figure design

Figure 5:

D. W. – conception, supervision, data analysis, figure design
S. Y. – conception, performance of the experiments, data analysis
K.B. – DNA construct generation and quality control, figure design

Figure 6:

D. W. – conception, supervision, data analysis, figure design
S. Y. – conception, performance of the experiments, data analysis
K.B. – DNA construct generation and quality control, figure design

Signed for and on behalf of the Author(s):	Print Name:	Date:
[signature]	David Wallach	8.12.21
